# BAP1 complex promotes transcription by opposing PRC1-mediated H2A ubiquitylation

**DOI:** 10.1038/s41467-018-08255-x

**Published:** 2019-01-21

**Authors:** Antoine Campagne, Ming-Kang Lee, Dina Zielinski, Audrey Michaud, Stéphanie Le Corre, Florent Dingli, Hong Chen, Lara Z. Shahidian, Ivaylo Vassilev, Nicolas Servant, Damarys Loew, Eric Pasmant, Sophie Postel-Vinay, Michel Wassef, Raphaël Margueron

**Affiliations:** 1 Institut Curie, Paris Sciences et Lettres Research University, Sorbonne University, 75005 Paris, France; 2 INSERM U934/CNRS UMR3215, 75005 Paris, France; 3 INSERM U900, Mines ParisTech, 75005 Paris, France; 4 Institute of Functional Epigenetics, Helmholtz Zentrum München, Neuherberg, 85764 Germany; 5 Department of Molecular Genetics Pathology, Cochin Hospital, HUPC AP-HP, EA7331, Faculty of Pharmacy, University of Paris Descartes, Paris, 75014 France; 6 Département d’Innovation Thérapeutique et Essais Précoces, INSERM U981, Gustave Roussy, Université Paris-Saclay, Villejuif, F-94805 France

## Abstract

In *Drosophila*, a complex consisting of Calypso and ASX catalyzes H2A deubiquitination and has been reported to act as part of the Polycomb machinery in transcriptional silencing. The mammalian homologs of these proteins (BAP1 and ASXL1/2/3, respectively), are frequently mutated in various cancer types, yet their precise functions remain unclear. Using an integrative approach based on isogenic cell lines generated with CRISPR/Cas9, we uncover an unanticipated role for BAP1 in gene activation. This function requires the assembly of an enzymatically active BAP1-associated core complex (BAP1.com) containing one of the redundant ASXL proteins. We investigate the mechanism underlying BAP1.com-mediated transcriptional regulation and show that it does not participate in Polycomb-mediated silencing. Instead, our results establish that the function of BAP1.com is to safeguard transcriptionally active genes against silencing by the Polycomb Repressive Complex 1.

## Abstract (web summary)

In *Drosophila*, the Calypso–ASX complex catalyzes H2A deubiquitination and aids Polycomb in transcriptional silencing. Here the authors show that the orthologous complex, BAP1.com, promotes gene activation by counteracting PRC1-mediated gene silencing.

## Introduction

BRCA1-associated protein 1 (BAP1) was initially characterized as a nuclear deubiquitinase regulating the function of BRCA1^1^. Subsequent work suggested that BAP1 does in fact interact with BRCA1-associated RING domain 1 (BARD1) and regulates its ubiquitination^2^. A variety of proteins have since been reported to interact with BAP1, including transcription factors (YY1, FOXK1/2), chromatin binders and modifiers (ASXL1/2/3, KDM1B, OGT1), the cell cycle regulator HCFC1 and DNA repair proteins (MBD5/6)^3–9^. BAP1 enzymatic activity has been shown to regulate the ubiquitination of various proteins including gamma-tubulin^10^, INO80^11^, and BRCA1^1^. Accordingly, BAP1 participates in diverse cellular processes, such as transcriptional regulation and the DNA damage response. However, the precise function of BAP1 in transcriptional regulation remains elusive. Some studies have reported that BAP1 acts as a transcriptional activator while others have suggested that it is required for gene silencing^3,4,12^.

In parallel to its characterization in mammals, studies in *Drosophila* identified the BAP1 ortholog Calypso as a novel Polycomb protein^13,14^. The Polycomb Group (PcG) of proteins is essential for the maintenance of gene repression, most prominently at developmentally regulated genes. Consequently, altering PcG function affects key cellular processes such as cell fate determination, cell proliferation, and genomic imprinting^15^. Two Polycomb complexes have been well characterized thus far: Polycomb Repressive Complex 1 (PRC1) and PRC2. PRC2 catalyzes di- and trimethylation of histone H3 on lysine 27 (H3K27me2/3), whereas PRC1 acts through chromatin compaction and monoubiquitination of histone H2A on lysine 119 (H2AK119ub1)^16,17^. Conserved from *Drosophila* to mammals, the activity of these complexes is necessary for maintaining transcriptional silencing of their target genes. The importance of H2AK119ub1 in Polycomb silencing has recently been called into question in *Drosophila*^18^, as well as in mouse models^19^. Nonetheless, recent studies suggest that this mark participates in stabilizing PRC2 binding to chromatin^20,21^.

*Drosophila* Calypso was found to partner with the Polycomb protein Additional Sex Combs (ASX) into a novel Polycomb complex termed Polycomb Repressive DeUBiquitinase (PR-DUB) complex. PR-DUB has been shown to catalyze deubiquitination of H2AK119ub1, opposite to the activity of PRC1^22^. The interaction between BAP1 and homologs of ASX (ASXL1/2/3 proteins) is conserved in mammals, as well as the H2AK119 DUB activity of BAP1^13,23^. How the antagonistic activities of Calypso/BAP1 and PRC1 converge to maintain transcriptional silencing remains enigmatic. Further, the link between PR-DUB and the Polycomb machinery is still controversial. Some studies have reported that ASXL1 interacts with PRC2 and is required for its recruitment^24–28^ while others have suggested an antagonism between BAP1 and PRC2^29–31^. A clear picture of the function of BAP1 and ASXL proteins is still lacking. Understanding the function of PR-DUB is all the more important in view of the tumor-suppressive functions of BAP1 in several cancer types including uveal melanoma, mesothelioma, and clear-cell renal cell carcinoma and of ASXL proteins in hematologic malignancies^2,32^.

In this study, we use biochemical, genome editing, and genome-wide methods to address the function of BAP1 in transcriptional regulation and its relationship to the Polycomb machinery. We show that the ASXLs are mandatory partners of BAP1 and are required for its stability and enzymatic activity. At the functional level, the complex formed with BAP1 (BAP1.com) is required for efficient transcription of many developmental genes. Accordingly, BAP1 appears to be largely dispensable for maintaining silencing of Polycomb target genes and in fact opposes PRC2-mediated silencing at a number of genes. The majority of BAP1-regulated genes, however, are not under regulation by PRC2, suggesting that the function of BAP1 in promoting transcription does not reflect an obligate antagonism with PRC2. We show that BAP1 is required upon transcriptional stimulation, as observed after retinoic acid (RA) treatment, a function that is shared with the CREBBP and SMARCB1 transcriptional co-activators. A general role in regulating gene expression is supported by the conspicuous colocalization between BAP1.com and RNA polymerase 2. Mechanistically, we show that BAP1’s function depends on its deubiquitinase (DUB) activity and that BAP1 is functionally inert in the absence of H2AK119ub1. Our integrative analysis uncovers an essential function for BAP1.com as a transcriptional co-activator, acting by locally antagonizing PRC1 activity.

## Results

### Loss of BAP1 alters the expression of developmental genes

**Fig. 1:**
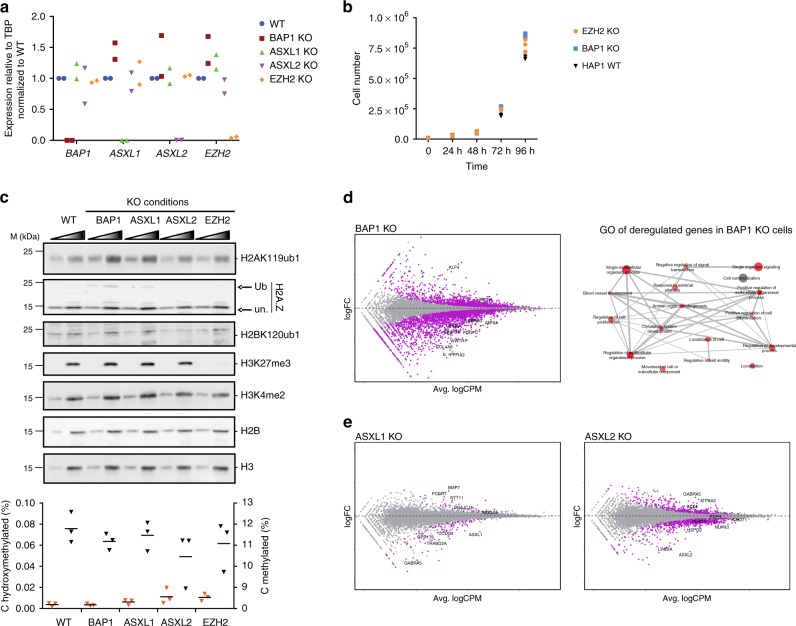
Functional consequences of loss of BAP1, ASXL1, or ASXL2 on chromatin and gene expression. **a** RT-qPCR analysis of *BAP1*, *ASXL1*, *ASXL2*, and *EZH2* expression in the different KO conditions indicated on top. *n* = 2. **b** Proliferation curve of wild-type, *EZH2* KO and *BAP1* KO HAP1 cells. *n* = 3. **c** Top, western blot analysis of acid extracted histones with antibodies directed against various histone modifications (as indicated on the right) in the different cell lines indicated on top, M  molecular weight. A two-point titration (1:2.5 ratio) is shown for each condition, ub  ubiquitinated, un unmodified. Bottom, analysis of cytosine methylation (blue triangles) or hydroxymethylation (red triangles) in the KO conditions indicated at the bottom. Horizontal bars indicate the mean. *n* = 3. **d** Left panel: scatterplot showing log2 fold-change (logFC) expression between wild-type and *BAP1* KO cells versus average log2 counts per million (logCPM). Differentially expressed genes (DEGs) in *BAP1* KO cells are highlighted in purple. Right panel: representation of the non-redundant most enriched GO terms within the DEGs in *BAP1* KO cells. **e** Scatterplots as in **d**, showing gene expression changes in *ASXL1* and *ASXL2* KO cells

In order to comprehend the role of BAP1 in transcriptional regulation, we generated knockouts (KOs) for *BAP1*, *ASXL1*, *ASXL2*, and *EZH2*, the main catalytic subunit of PRC2, in human HAP1 cells, a model cell line that is nearly haploid and thus particularly amenable to genome editing^33^. KOs result from the insertion of a STOP cassette that interrupts transcription and translation of the target gene^34^ and were validated by reverse transcription-quantitative polymerase chain reaction (RT-qPCR, Fig. 1a). In contrast to many cell lines where the knockdown of BAP1 severely compromises proliferation (Supplementary Fig. 1a), we found that BAP1 is dispensable for proliferation in HAP1 cells, thus providing a suitable system for studying its mechanism of action (Fig. 1b). Cellular fractionation confirmed the tight association of BAP1 with chromatin, as shown by its enrichment both in the soluble and insoluble fractions (Supplementary Fig. 1b), which prompted us to further investigate its chromatin-modifying activity. Western blot analysis of various histone marks showed that BAP1 loss is associated with an approximate twofold increase in total H2A ubiquitination levels, as well as a parallel increase in H2A.Z ubiquitination, with no effect on H2B ubiquitination (Fig. 1c, top panel). *ASXL1* and *ASXL2* KO led to a modest increase in H2A/H2A.Z ubiquitination. We did not observe any global effect upon KO of *BAP1*, *ASXL1*, or *ASXL2* on other histone marks such as H3K4me2 and H3K27me3 or on DNA modifications (Fig. 1c, bottom panel).

We then performed RNA-seq to analyze the transcriptome of wild-type and KO cell lines. The inactivation of BAP1 resulted in dramatic changes in gene expression (*n* = 1893 differentially expressed (DE) genes, false discovery rate (FDR) < 0.05 and absolute log2 fold-change > 1; Fig. 1d, left panel). Strikingly, the majority of affected genes were downregulated in *BAP1* KO cells (*n* = 1370), suggesting an involvement in gene activation rather than silencing. Gene ontology (GO) analysis revealed enrichment for a variety of biological processes ranging from broad terms such as cell communication, signaling, or regulation of proliferation to more specific terms such as blood vessel development (Fig. 1d, right panel). Several terms related to development were also enriched, consistent with the reported function of the BAP1 ortholog in *Drosophila*^13^. Inactivation of either ASXL protein also leads to preferential downregulation of genes (85 downregulated genes of 112 total DE genes in *ASXL1* KO cells and 262 downregulated genes of 406 DE genes in the *ASXL2* KO cells; Fig. 1e, also see Supplementary Fig. 1c for heatmaps of the top 100 DE genes in each KO condition) but has a much milder effect on gene expression than observed for the *BAP1* KO. Further, loss of ASXL1 or ASXL2 does not affect the chromatin localization of BAP1 (Supplementary Fig. 1b). This result suggests either that BAP1 can function independently of its interaction with ASXL proteins or that the ASXLs are largely redundant.

### The ASXL/BAP1 core complex is conserved in mammals

**Fig. 2:**
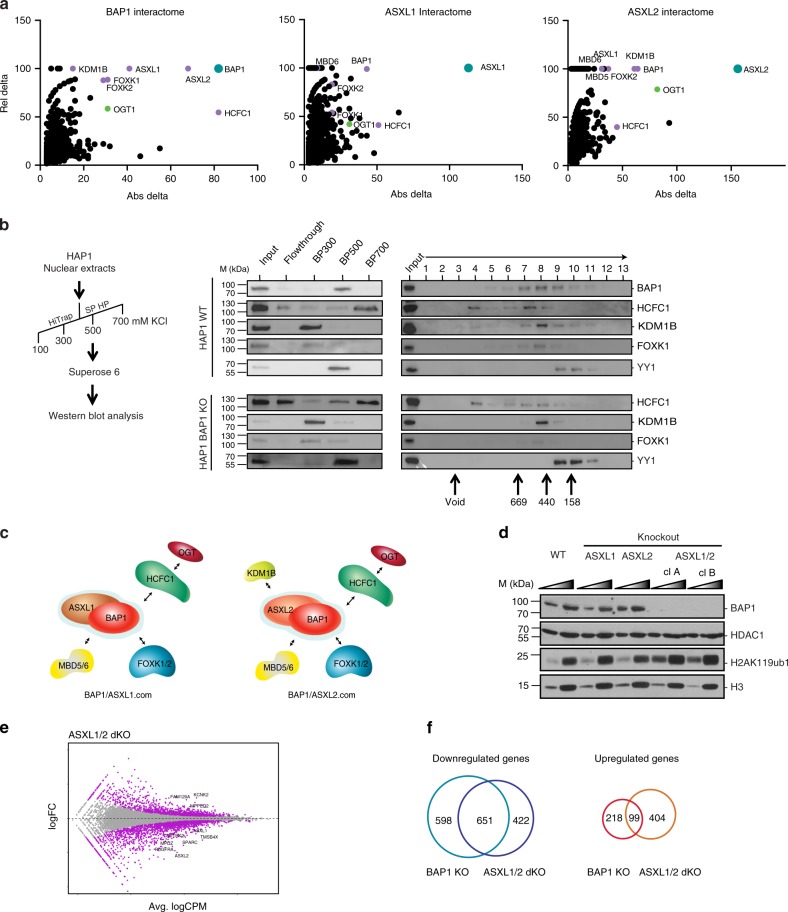
BAP1.com core complex and associated factors. **a** Mass spectrometry analysis of HeLa cells overexpressing Flag-tagged versions of BAP1, ASXL1, and ASXL2. Graphs represent proteins relative to their absolute (Abs) and relative (Rel) delta compared with mass spectrometry analysis of empty vector expressing cells. Absolute delta is the absolute difference between distinct peptides identified in sample and control; relative delta is the ratio of absolute delta versus the sum of distinct peptides identified in sample and control. **b** Elution patterns of HAP1 WT and HAP1 *BAP1*-KO nuclear extracts following the purification scheme indicated in the left panel and monitored by western blot with the indicated antibodies. Middle pattern is a representative elution pattern (step elution with increased salt concentration) on a cation exchange column (SP-HP, GE). Right panel is a representative elution pattern on a size-exclusion column (Superose 6, PC3.2/30, GE)*. Underneath is the correspondent*. **c** Schematic of BAP1.com core complex and associated factors depending on the ASXL paralog present. **d** Western blot analysis of BAP1 and H2AK119ub1 in the different KO conditions indicated above. Two independent clones of *ASXL1/2* dKO cells are shown. HDAC1 and H3 serve as nuclear and histone protein loading control respectively. A two-point titration (1:2.5 ratio) is shown for each condition. **e** Scatterplot showing log2 fold-change (logFC) expression between wild-type and *ASXL1/2* dKO cells as a function of average log2 counts per million (logCPM). Differentially expressed genes in *ASXL1/2* dKO cells are highlighted in purple. **f** Venn diagram showing the overlap between genes downregulated^60^ or upregulated (right) in *BAP1* KO and *ASXL1*/2 dKO cells

To better understand the function of BAP1, we sought to determine whether it is part of a stable complex and, if so, with which partners. Purification of Calypso from *Drosophila* embryos revealed that it forms a heterodimeric complex with ASX^13^. Although the interaction between BAP1 and ASXL proteins is conserved^13,23,35^, additional partners were reported in the mammalian complex^3,4,9^. We overexpressed FLAG-tagged versions of BAP1, ASXL1, or ASXL2 in HeLa cells (Supplementary Fig. 2a), followed by immunoprecipitation and mass spectrometry. The results confirmed previous reports, notably the identification of ASXL1/2, FOXK1/2, HCFC1, and KDM1B as partners of BAP1 (Fig. 2a). The ASXL1 and ASXL2 interactomes were similar with the exception of KDM1B, which is specifically pulled down by ASXL2. Of note, similar results were obtained for the BAP1 interactome in Uveal Melanoma MP41 cells (Supplementary Fig. 2b), suggesting that BAP1.com composition is not cell-type dependent.

We then sought to determine whether BAP1 partners are all present in a single complex or if BAP1 is engaged in distinct protein complexes. To this end, we analyzed the elution pattern of BAP1 partners by ion exchange chromatography followed by size-exclusion chromatography (SEC) (Fig. 2b). The first purification step (ion exchange) revealed that the HCFC1, FOXK1, and KDM1B generally elute independently of BAP1, which is almost exclusively found in the 500 mM salt fraction, suggesting that only a portion of each of these proteins is engaged in a complex with BAP1. In the second purification step (SEC), we observed that BAP1 elutes with a molecular weight of approximately 500 kDa, along with with HCFC1, FOXK1, and KDM1B. In contrast, YY1 elutes later and only partially overlaps with BAP1. To know whether the co-elution between BAP1 and its cofactors reflects the assembly of these proteins into a complex, we repeated the experiment with nuclear extract from BAP1 KO cells. The elution pattern from cation exchange did not reveal any major changes; we therefore continued with SEC. The elution patterns of HCFC1, KDM1B, and FOXK1 remain unchanged, indicating that the co-elutions observed with BAP1 in the wild-type extract do not reflect the formation of a stable complex (Fig. 2b). Considering the previously established tight interaction between BAP1 and the ASXLs^13,23,35^, our results suggest that BAP1 and ASXL proteins form a core complex (BAP1.com), which engages in transient interactions with additional partners such as FOXK1/2, HCFC1, or KDM1B (Fig. 2c). This model predicts that immunoprecipitation of any one of these transient partners would consistently retrieve the core complex but not necessarily other transient partners. Indeed, KDM1B immunoprecipitation from HeLa cells pulls down ASXL2 and BAP1 (as well as NSD3, which is part of a distinct complex with KDM1B) but none of the other transient partners (Supplementary Fig. 2c).

Given the role of ASXL1 and ASXL2 in driving BAP1-associated complex composition but the modest effect of their individual deletion on transcription, we sought to investigate their potential redundancy in BAP1-mediated H2A deubiquitination and transcriptional regulation. To address this question, we generated a double *ASXL1*/*ASXL2* KO. Of note, *ASXL3* is not expressed in wild-type HAP1 cells nor in *ASXL1/2* double KO cells (Supplementary Fig. 2d). We first evaluated the effect of this double KO on chromatin regulation and observed a robust increase in H2A ubiquitination levels similar to those observed in the *BAP1* KO (Fig. 2d). This result is consistent with the fact that interaction with the ASXLs is required for BAP1 enzymatic activity (Supplementary Fig. 2e). Notably, loss of ASXL1 and ASXL2 also led to a dramatic reduction in BAP1 protein levels (Fig. 2d), whereas *BAP1* transcript levels were unaffected (Supplementary Fig. 2d). Thus, ASXLs are not only necessary for the enzymatic activity of BAP1 but also for protein stability in vivo. Consistent with this effect on BAP1 protein accumulation, transcriptome analysis of *ASXL1/2* dKO cells revealed a major impact on gene expression (Fig. 2e). Most DE genes were downregulated (70%, 1073 downregulated genes out of 1576 total DE genes, also see Supplementary Fig. 2f for heatmaps of the top 100 DE genes) and there was a large overlap between genes downregulated in *BAP1* KO and *ASXL1/2* dKO cells (Fig. 2f, left). In comparison, the overlap between upregulated genes was much less pronounced (Fig. 2f, right), supporting the idea that the main role of BAP1.com is to promote transcription. Altogether, these results show that ASXL proteins are mandatory and redundant partners of BAP1.

### BAP1 does not participate in Polycomb-mediated silencing

**Fig. 3:**
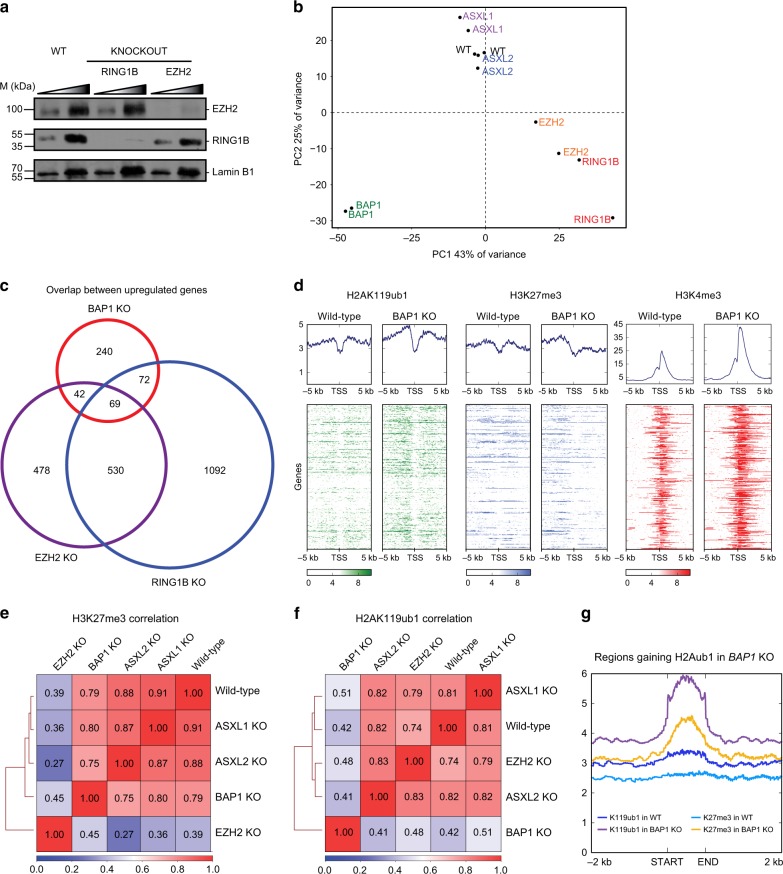
BAP1.com is dispensable for Polycomb-mediated silencing. **a** Western blot analysis of EZH2 and RING1B in wild-type (WT), *RING1B* KO, and *EZH2* KO cells. Lamin B1 is used as a loading control. A two-point titration (1:2 ratio) is shown for each condition. **b** PCA analysis of WT, *RING1B*, *EZH2*, *ASXL1*, *ASXL2*, and *BAP1* KO transcriptome. **c** Venn diagram showing the overlap between genes upregulated in *BAP1*, *EZH2*, or *RING1B* KO cells. **d** Heatmaps showing H2AK119ub1, H3K27me3, and H3K4me3 distribution in a −5/ + 5 kb window around the transcription start site (TSS) of genes upregulated in *BAP1* KO cells in wild-type and *BAP1* KO cells. Corresponding average profiles are plotted on top of each heatmap. **e**, **f** Correlation heatmap of H3K27me3 (**e**) and H2AK119ub1 (**f**) distribution between wild-type cells and the different KO conditions indicated. **g** Plots showing average enrichment of H2AK119ub1 and H3K27me3 in wild-type and *BAP1* KO cells at regions that gain H2AK119ub1 upon BAP1 loss

The data presented above suggest that the main role of BAP1 is to positively regulate transcription. While in agreement with several previously published studies, our findings contrast with a number of reports suggesting that BAP1 and ASXL proteins participate in Polycomb-mediated silencing^24–28^. To formally investigate the interplay between BAP1 and Polycomb proteins, we analyzed the consequences of BAP1 loss in conjunction with loss of RING1B and EZH2, key members of PRC1 and PRC2, respectively (Fig. 3a).

As expected, the main impact of inactivating either *RING1B* or *EZH2* is the transcriptional upregulation of a large set of genes (Supplementary Fig. 3a, b). The GO terms for the DE genes (either up or down regulated) in EZH2 KO cells partially overlap the categories observed upon BAP1 KO, including those related to signaling or development (Supplementary Fig. 3c and Fig. 1d, right panel). The terms associated with genes DE in the absence of RING1B are broader, which might reflect a less developmental specific function of PRC1 (Supplementary Fig. 3d). To further compare transcriptional changes between all KO conditions, we performed principal component analysis (PCA, Fig. 3b). Along PC1, most of the variance is driven by differences in BAP1 and polycomb machinery, again suggestive of distinct gene regulatory functions. As the Polycomb machinery is involved in gene silencing, we investigated the overlap between genes upregulated upon KO of *EZH2* or *RING1B* and KO of *BAP1*. As shown in Fig. 3c, only a minority of the genes regulated by PRC1 and/or PRC2 becomes upregulated upon loss of BAP1. To determine whether this limited overlap reflects a synergistic action of BAP1 with the Polycomb machinery, we investigated chromatin changes occurring at genes upregulated in *BAP1* KO. If BAP1 functions together with the Polycomb machinery to maintain transcriptional silencing at a subset of Polycomb target genes, loss of BAP1 is expected to result in a decrease in the Polycomb-mediated chromatin signature. However, chromatin immunoprecipitation (ChIP) followed by deep sequencing (ChIP-seq) did not reveal a decrease in Polycomb histone marks H2AK119ub1 or H3K27me3 at these genes in *BAP1* KO cells (Fig. 3d). In fact, H2AK119ub1 increased, possibly reflecting the more global increase of the mark caused by loss of BAP1 (see further below). As expected, transcriptional upregulation corresponded with marked increase in H3K4me3, a histone mark deposited preferentially near the 5ʹ ends of transcriptionally active genes. These results suggest that transcriptional upregulation occurring upon BAP1 loss is not caused by impaired Polycomb-mediated silencing. Instead, these gene expression changes may be secondary effects of widespread transcriptional downregulation.

We then analyzed the genome-wide distribution of the Polycomb-specific histone marks H3K27me3 and H2AK119ub1. Previous studies have reported a crucial role for the ASXLs in H3K27me3 deposition^24–28^, but analysis of H3K27me3 revealed a high correlation in the genome-wide localization of the mark between wild-type, *BAP1*, *ASXL1*, and *ASXL2* KO cells, suggesting that loss of the ASXL proteins does not globally affect H3K27me3 distribution (Fig. 3e see also Supplementary Fig. 3e). This analysis, together with the lack of global change in H3K27me3 abundance in *BAP1*, *ASXL1*, or *ASXL2* KO cells as gauged by western blot (Fig. 1c), rules out an essential role for BAP1 and ASXL proteins in PRC2 function. In contrast, the distribution of H2AK119ub1 was significantly altered in BAP1 KO cells (Fig. 3f). Differential analysis of H2AK119ub1 signal between BAP1 KO and wild-type cells revealed widespread gains upon loss of BAP1 (12,388 regions) and much less depleted regions (3456), in keeping with the global increase of the mark seen by western blot analysis (Fig. 1c). H2AK119ub1 gains localized throughout the genome with an approximate twofold enrichment at putative promoter and enhancer regions over random peaks (Supplementary Fig. 3f). Interestingly, consistent with the known interplay between PRC1 and PRC2, gains of H2AK119ub1 were accompanied by an increase of H3K27me3 (Fig. 3g). Altogether, these data establish that BAP1.com does not act in synergy with the Polycomb machinery to maintain gene silencing but, instead, that its activity might restrain Polycomb enzymatic activity.

### PRC2-antagonistic and -independent role of BAP1

**Fig. 4:**
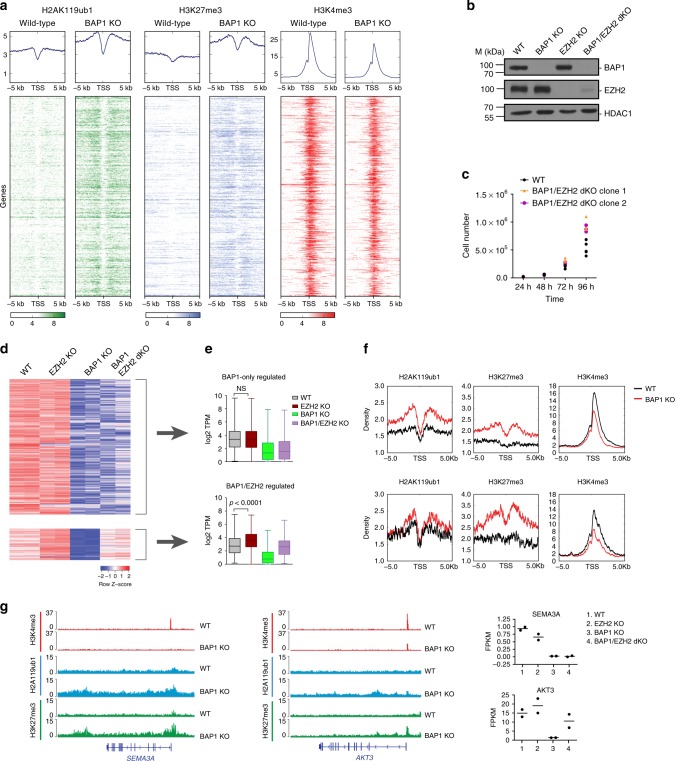
BAP1.com promotes transcription independently of an antagonism with the PRC2 complex. **a** Heatmaps showing H2AK119ub1, H3K27me3, and H3K4me3 distribution in a −5/ + 5 kb window around the transcription start site (TSS) of genes downregulated in *BAP1* KO cells in wild-type and BAP1 KO cells. Corresponding average profiles are plotted on top of each heatmap. **b** Western blot analysis of BAP1 and EZH2 in single and double *BAP1/EZH2* KO cells. HDAC1 is used as a loading control. **c** Dot plot showing proliferation of wild-type cells and two independent clones of *BAP1/EZH2* dKO cells. *n* = 3. **d** Heatmap of gene expression (*Z*-scores) in the different genotypes. Top: BAP1-only-regulated genes, and bottom: BAP1- and EZH2-regulated genes, TSS = transcription start site. **e** Box-plots (median, lower, and upper quartiles, lowest and highest values) of log2 transcript per million (TPM) expression values of BAP1-only-regulated genes (top) and BAP1/EZH2-regulated genes^61^ in the different conditions as indicated. Result of the Mann–Whitney test on the *EZH2* KO versus wild-type comparison is indicated. **f** Plot showing average enrichment of H2AK119ub1, H3K27me3, and H3K4me3 in a −5/ + 5 kb around the TSS for BAP1-only-regulated genes (top) and BAP1/EZH2-regulated genes^61^. **g** Example snapshots of H2AK119ub1, H3K27me3, and H3K4me3 enrichment in WT and *BAP1* KO cells at a BAP1-only-regulated gene and a BAP1/EZH2-regulated gene (middle). Expression values of the corresponding genes across WT, *EZH2*, *BAP1*, and *BAP1/EZH2* KO conditions as detected in corresponding RNA-seq data are shown on the right. Horizontal bars indicate the mean expression

As shown above, the major impact of loss of BAP1 is the downregulation of gene expression accompanied by widespread gains of H2AK119ub1 and H3K27me3. To investigate whether gains of Polycomb marks and transcriptional changes following BAP1 loss are linked, we first analyzed changes in chromatin composition at genes that are downregulated in the absence of BAP1 (Fig. 4a). As expected, transcription-associated H3K4me3 decreased concomitantly with transcriptional downregulation. In principle, increased levels of the H2AK119ub1 and H3K27me3 repressive marks could be a direct consequence of loss of BAP1 deubiquitinase activity or could be a secondary event caused by transcriptional downregulation^36^. To discern between these two possibilities, we genetically inactivated *EZH2* in *BAP1* KO cells (Fig. 4b) and assessed whether *EZH2* deletion can restore expression of BAP1-regulated genes. Of note, *EZH2* deletion in cells already KO for *BAP1* did not impair proliferation (Fig. 4c), consistent with recent evidence challenging the reported synthetic lethal relationship between EZH2 inhibition and BAP1 inactivation^29,37^. Of the 913 genes downregulated upon *BAP1* KO, a large majority (741 genes) remain silent in the *BAP1/EZH2* double KO (Fig. 4d, top heatmap), whereas the expression of a minority (172 genes) is increased in the double KO compared with BAP1 single KO cells (Fig. 4d, bottom heatmap). This set of genes is also upregulated upon deletion of *EZH2* in a *BAP1* wild-type context, indicative of a balanced antagonistic regulation between BAP1 and EZH2 (Fig. 4d, e). Moreover, all genes downregulated in the absence of BAP1 gain H3K27me3 upon *BAP1* KO (Fig. 4f, see also Fig. 4g and Supplementary Fig. 4a, b for specific examples), suggesting that such a gain is a consequence rather than a cause of transcriptional downregulation. Thus, although BAP1 and PRC2 act in an opposite fashion at a number of genes, BAP1 promotes gene expression in a manner that is largely independent of an antagonism with the PRC2 complex.

### Similarities between BAP1.com and general co-activators

**Fig. 5:**
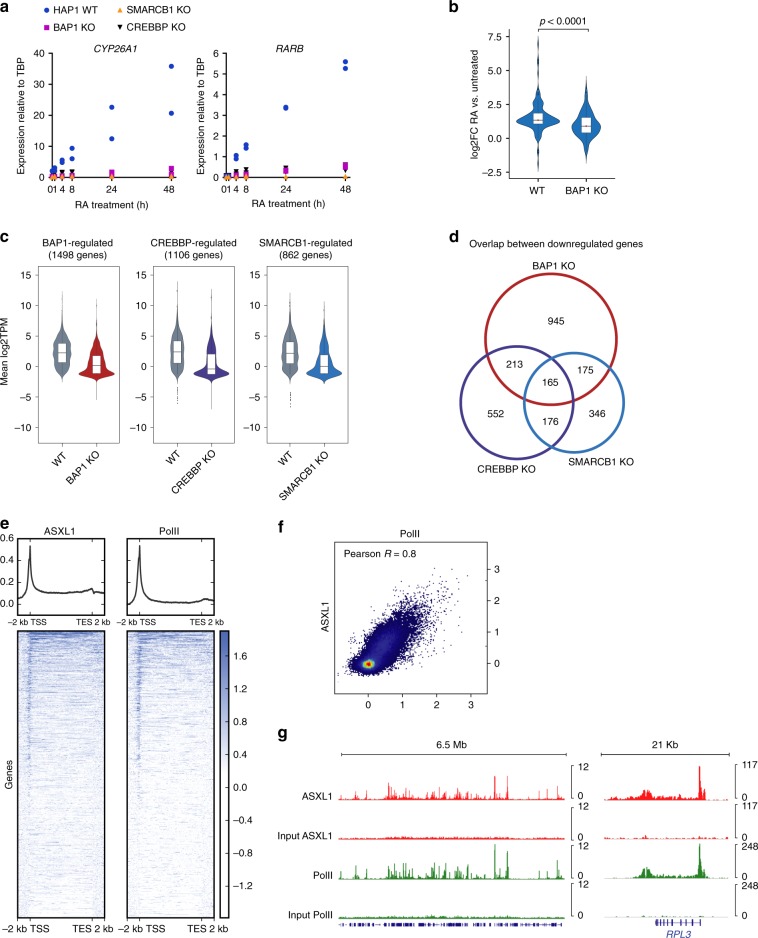
Comparison of BAP1 and the CREBBP and SMARCB1 co-activator proteins. **a** RT-qPCR analysis of *CYP26A1* and *RARB* expression following RA treatment at different time-points in wild-type or *BAP1*, *CREBBP*, and *SMARCB1* KO cells. *n* = 2. **b** Violin plots showing log2 fold-change expression of RA-responsive genes (*n* = 114 genes, see text for details) in wild-type and BAP1 KO cells. *P*-value from the Mann–Whitney test is shown. **c** Violin plots showing log2TPM expression of BAP1- CREBBP-, and SMARCB1-regulated genes in wild-type (WT) or in the respective KO conditions. **d** Venn diagram showing overlap between genes that are downregulated in *BAP1*, *CREBBP*, and *SMARCB1* KO cells. **e** Heatmaps showing ASXL1 and RNA PolII density around the TSS and termination end site (TES) (including 2-kb upstream and downstream) scaled to an equivalent 10 kb in human HEK-293 cells. Corresponding average profiles are plotted above each heatmap. **f** Scatterplot showing PolII versus ASXL1 enrichment around the TSS (including 2 kb upstream and downstream) at all annotated genes. Pearson correlation coefficient is displayed. **g** Snapshots of ASXL1 and RNA PolII enrichment at representative regions. The input is displayed below each corresponding ChIP-seq experiment

Having analyzed the impact of loss of BAP1.com on steady-state gene transcription, we next sought to examine its role in transcriptional activation in response to a transcriptional stimulus. Considering previous reports suggesting that BAP1 may modulate nuclear receptor-mediated gene regulation^38,39^, we investigated whether deletion of *BAP1* would affect response to RA treatment. We initially performed a time-course analysis of two of the best-characterized direct RA transcriptional targets: *RARβ* and *CYP26A1* (Fig. 5a). In contrast to the above-mentioned reports that suggest a repressive role for BAP1.com at RA target genes, we observed that activation of both *RARβ* and *CYP26A1* was severely compromised in *BAP1* KO cells. Of note, loss of BAP1 did not affect the protein levels of RARα, a major RA-binding nuclear receptor (Supplementary Fig. 5a). To determine whether these results reflect a general role for BAP1 in the transcriptional response to RA, we analyzed the transcriptome of WT and *BAP1* KO cells in response to RA treatment (after 24 h). Nearly twice as many genes were significantly activated upon RA treatment in WT than in *BAP1* KO cells (*n* = 88 versus *n* = 47, FDR < 0.05 and absolute log2 fold-change > 1, Supplementary Fig. 5b). Furthermore, analyzing the entire set of genes activated in either condition, we found that the response to RA is significantly attenuated in *BAP1* KO cells (Fig. 5b), demonstrating that BAP1 is required for optimal RA-mediated transcriptional activation.

To further investigate the hypothesis that BAP1 functions as a transcriptional co-activator, we compared transcriptional defects occurring in *BAP1* KO cells and KOs of *SMARCB1* (Supplementary Fig. 5c), which encodes an essential member of the BAF chromatin-remodeling complex, and *CREBBP*, which encodes a histone acetyltransferase. Both KO cell lines were viable, although they appeared less healthy than the wild-type counterpart. As with loss of BAP1, loss of SMARCB1 and CREBBP severely impaired RA-mediated transcriptional activation of *RARβ* and *CYP26A1* (Fig. 5a). To determine to what extent BAP1, CREBBP, and SMARCB1 affect transcription, we compared the transcriptome of HAP1 cells mutated for each of these genes (this study and^40^). As shown in Fig. 5c, each KO leads to the downregulation of a similar number of genes (BAP1: 1498 genes, CREBBP: 1106 genes and SMARCB1: 862 genes). We next compared the overlap between genes regulated by the three proteins. Although RNA-seq for each KO condition was performed in different laboratories, there was a significant overlap between genes downregulated upon loss of BAP1, CREBBP, or SMARCB1 (Fig. 5d, *p* < 0.0001 for all three comparisons). Nonetheless, the majority of downregulated genes remain specific to each respective KO, indicating that BAP1, CREBBP, and SMARCB1 each regulate a distinct set of genes.

To determine the level of specificity of gene recruitment, we compared BAP1.com localization at chromatin to the localization of transcription machinery. We obtained previously published ChIP-seq data for ASXL1^9^ and RNA-PolII (ENCODE) in human HEK-293 cells, and BAP1^4^ and RNA-PolII (ENCODE) in mouse bone marrow-derived macrophages. Analysis of ASXL1/PolII and BAP1/PolII enrichment revealed a marked correlation between RNA-PolII and BAP1.com profiles, both in terms of enrichment intensity and profile along the gene body (Fig. 5e, f and Supplementary Fig. 5d, e and ChIP-seq screenshots in Fig. 5g and Supplementary Fig. 5f). This result strongly supports a role for BAP1.com as a general co-activator and suggests that functional differences between BAP1 and other co-activators are due to gene-specific requirements for their respective enzymatic activities during transcriptional activation, rather than gene-specific targeting. Together, these data provide compelling evidence that BAP1.com functions as a general transcriptional co-activator.

### PRC1 is epistatic to BAP1

Finally, we sought to understand how BAP1 exerts its function on transcription. In principle, BAP1 could act through its enzymatic activity to modify proteins that modulate transcription, and/or by recruiting factors that participate in promoting transcription. Several reported BAP1 substrates such as H2AK119ub1, BARD1, HCFC1, and OGT could potentially mediate its function in gene activation^4–6,41^. HCFC1 is a transcriptional regulator and, in addition to modulating its ubiquitination, BAP1 has been suggested to contribute to its recruitment and/or regulation of its stability. More recently, BAP1 was suggested to participate in the recruitment of the MLL3 histone methyltransferase at enhancers by direct interaction with MLL3 PHD repeats^31^.

**Fig. 6:**
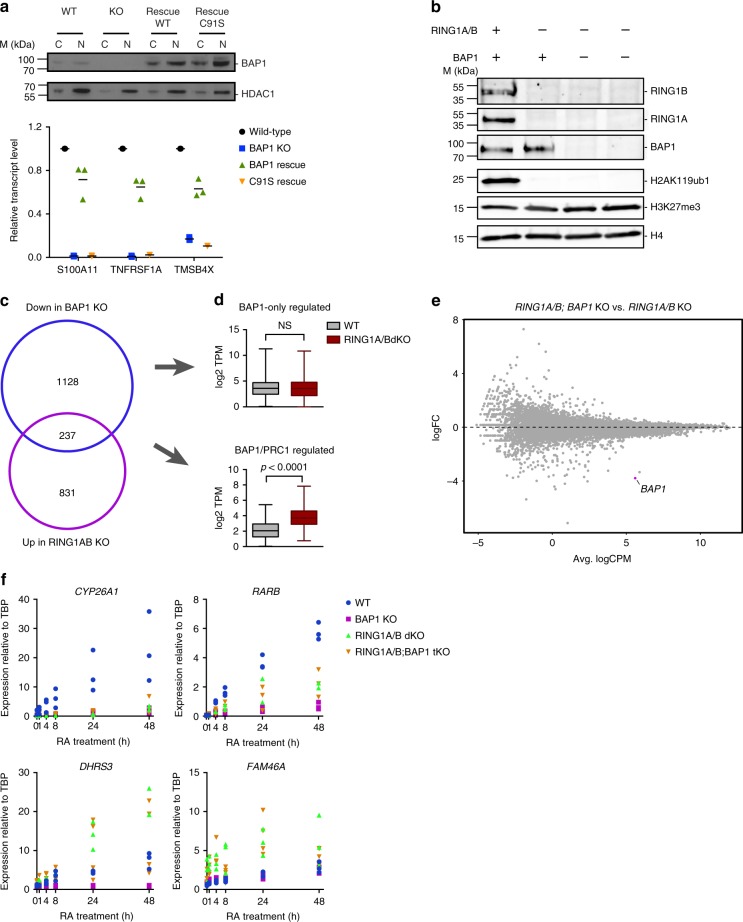
BAP1 function relies on its deubiquitinase activity against H2AK119ub1. **a** Reintroduction of wild-type or catalytically dead (C91S) BAP1 in *BAP1* KO cells. Top panel: western blot analysis of BAP1 expression in cytoplasmic (C) or nuclear (N) fractions. HDAC1 is used as a loading control. Bottom panel: RT-qPCR analysis of three BAP1-regulated genes in wild-type, *BAP1* KO and the two rescue conditions. Horizontal bars indicate the mean expression. *n* = 3. **b** Western blot analysis of RING1A, RING1B, BAP1, H2AK119ub1, and H3K27me3 in wild-type, *RING1A/B* double KO, or *RING1A/B*; *BAP1* triple KO HAP1 cells. H4 is used as a loading control. **c** Venn diagram showing the overlap between genes downregulated in *BAP1* KO or upregulated in *RING1A/B* dKO cells. **d** Box-plots (median, lower, and upper quartiles, lowest and highest values) of log2 transcript per million (TPM) expression values of BAP1-only-regulated genes (top) and BAP1/PRC1-regulated genes^61^ in WT and *RING1A/B* dKO cells. *P*-value of the Mann–Whitney test on WT versus *RING1A/B* dKO comparison is indicated. **e** Scatterplot showing log2 fold-change (logFC) expression between *RING1A/B* dKO and *RING1A/B; BAP1* tKO cells as a function of average log2 counts per million (logCPM). *BAP1*, the only differentially expressed genes is highlighted in purple. **f** RT-qPCR analysis of *CYP26A1*, *RARB*, *DHRS3,* and *FAM46A* expression following RA treatment at different time-points in wild-type or *BAP1*, *RING1A/B*, *RING1A/B;BAP1* KO cells

To determine which of these functions is critical for BAP1 activity, we first assessed if BAP1 function requires its DUB activity. For this purpose, we performed rescue experiments in *BAP1* KO cells, reintroducing either wild-type BAP1 or a catalytically dead version (BAP1 C91S). Both versions of BAP1 were re-expressed at a similar level, slightly higher than the original endogenous level (Fig. 6a, upper panel). Focusing on a selection of genes whose expression is dramatically reduced in the absence of BAP1, re-expression of wild-type BAP1 protein restores up to 75% of the wild-type levels of the transcripts while the C91S mutant is unable to rescue transcription of the tested genes (Fig. 6a, lower panel). Although based on a subset of genes, this analysis suggests that BAP1 catalytic activity is required for its function, consistent with previous studies^3,13^.

**Fig. 7:**
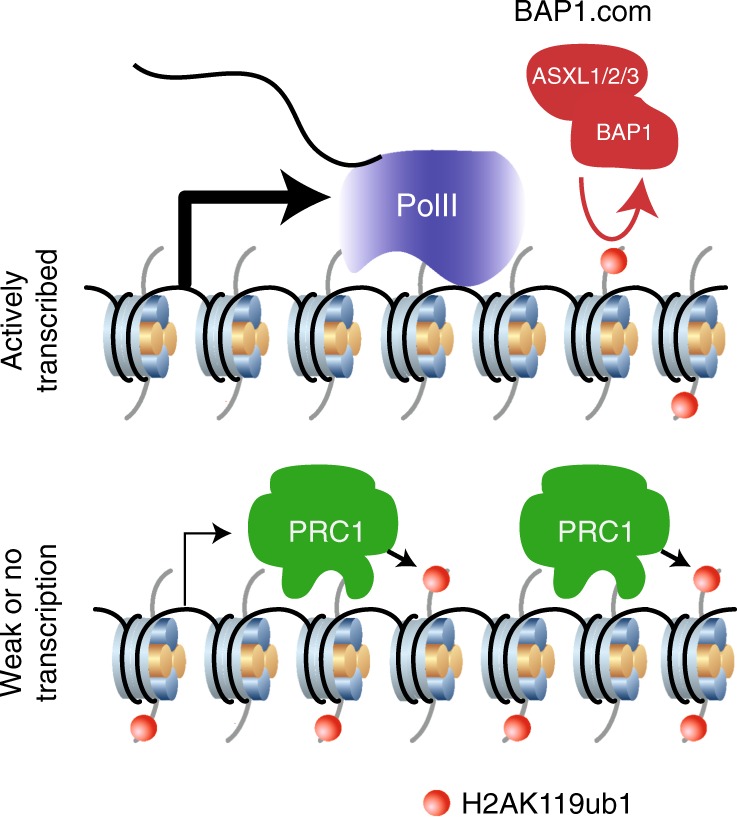
Model of BAP1.com function

We next sought to determine the relative contribution of H2AK119ub1 compared with other substrates to BAP1’s function. H2AK119ub1 is solely deposited by the PRC1 complex through its two paralogous enzymes RING1A and RING1B. We thus genetically inactivated both enzymes in HAP1 cells and then mutated BAP1 in the context of *RING1A/B* dKO cells. As expected, H2AK119ub1 was completely absent from both *RING1A/B* dKO and *RING1A/B;BAP1* tKO cells (Fig. 6b). Of note, proliferation of the two mutant conditions was unimpaired (Supplementary Fig. 6). We first assessed the overlap between genes positively regulated by BAP1 and negatively regulated by PRC1. As shown in Fig. 6c, a minority of genes, 17%, were under such opposite regulation. Analysis of BAP1-only-regulated genes as a group confirmed the absence of regulation by PRC1 (Fig. 6d) and also showed that this set of genes has an overall higher level of expression compared with BAP1/PRC1-regulated genes (Fig. 6d). To determine whether this BAP1-mediated transcriptional regulation nevertheless depends on PRC1-mediated H2AK119ub1, we assessed transcriptional changes in *RING1A/B*;*BAP1* tKO versus *RING1A/B* dKO. *BAP1* was the only DE transcript between the two mutant conditions (Fig. 6e), indicating that BAP1 is no longer functional in the absence of PRC1 activity. Consistently, transcriptional response to RA treatment was equivalent in *RING1A/B* dKO and *RING1A/B*; *BAP1 t*KO cells (Fig. 6f, transcriptional response to RA differs in *RING1A/B* dKO cells compared with WT cells). This result contrasts with the limited dependency of loss of BAP1 on the PRC2 complex (Fig. 4), and suggests that gain of H2AK119ub1 might inhibit transcription in a way that is partly independent of PRC2. Taken together, these results establish that PRC1 is epistatic to BAP1.com and that BAP1 promotes transcription by counteracting PRC1-mediated H2A ubiquitination (Fig. 7).

## Discussion

Despite their prominent roles as tumor suppressors, the biological functions of BAP1 and ASXL proteins remain poorly characterized. In this study, we performed extensive biochemical and genetic analyses in isogenic mutant cell lines to address the role of BAP1 in transcriptional regulation, the respective contributions of BAP1 and the ASXL proteins to the regulation of gene expression and the interplay between BAP1.com and the Polycomb machinery.

Both biochemical and genetic evidence indicate that BAP1 and ASXL proteins function together to regulate H2AK119ub1 and gene expression. Our biochemical analyses largely confirm the previously reported interactome of BAP1^9^ and further enable distinguishing transient interactors (FOXK1/2, MBD5/6, HCFC1, etc.) from the core complex composed of BAP1 and one ASXL paralog. With the exception of KDM1B, which appears to interact specifically with BAP1-ASXL2, the BAP1 interactome is essentially identical whether the complex forms around ASXL1 or ASXL2. This might reflect the fact that most of the interactions are mediated directly by BAP1, as suggested for HCFC1, YY1, OGT, and FOXK1/2^3,35^. Nonetheless, interaction with the ASXL paralogs is required for BAP1 enzymatic activity and protein stability. The overall interchangeability between the ASXLs explains the observation that knocking out a single paralog only mildly affects gene expression. An immediate implication of this finding concerns the tumor-type-specific spectrum of BAP1 and ASXL mutations. While previous studies have argued that this non-overlapping mutation spectrum might be the result of independent and sometimes opposite functions of BAP1 and ASXL proteins^24,29^, our findings instead suggest that, due to the redundancy among ASXL proteins, the loss of only one of them results in a much less severe disruption of BAP1.com function than loss of BAP1. Hence, we propose that the predominance of ASXL mutations in myeloid malignancies may be the result of a selective pressure aimed at only partially ablating BAP1 function, whereas loss of BAP1 in other malignancies would reflect a need for complete inactivation of the complex.

In contrast to the reported role of Calypso in *Drosophila*, our results argue against an implication of BAP1.com in the Polycomb machinery. Instead, the picture that emerges from this work is that the function of BAP1.com is to promote transcription by limiting PRC1 repressive activity. This property may not be surprising given that the activity of the complex is opposite to that of PRC1 in the regulation of H2A ubiquitination and that PRC1 and ASXL1 show rather distinct localization patterns^9^. The more limited dependency on PRC2 activity that we observed is likely the result of partly divergent functions between the two Polycomb complexes, and in particular the ability of PRC1 to be recruited and silence genes independently of PRC2^42,43^.

Our findings are also in line with previous studies using artificial recruitment of BAP1 to a transgene^3^, overexpressing hyperactive forms of ASXL1^44^ or focusing on individual loci^30^, which all support a role for BAP1 in gene activation. An obvious question arising from these results is whether BAP1 and Calypso have divergent functions or whether we can reconcile their proposed contributions to gene regulation in both species. First, it is noteworthy that there is no clear consensus as to whether the Calypso–Asx complex is generally involved in Polycomb-mediated silencing. Indeed, mutation of Asx in *Drosophila* leads to a complex phenotype that exhibits features of both Polycomb and Trithorax mutants^45^, a situation that is also found in *Asxl1* mutant mouse embryos^46^. Second, only a subset of Polycomb target genes was found to be aberrantly activated upon loss of Asx or Calypso^47^. Third, part of the difference might result from the extent to which BAP1 and Calypso regulate H2A ubiquitination. Although loss of Asx leads to an approximate 10-fold increase in the total levels of H2AK118ub1^22^, we only observe an approximate twofold increase in the level of the mark upon inactivation of either of BAP1 or ASXL1/2. This suggests that in *Drosophila*, the Calypso–Asx complex might have a more critical function in restricting PRC1 activity to its normal site of action. Aberrant deposition of H2AK119ub1 and consequently of H3K27me3 could indirectly impair Polycomb-mediated transcriptional silencing. Genome-wide functional studies analyzing the global consequences of loss of Asx or Calypso in flies should help clarify these questions and address whether PR-DUB and BAP1.com have similar functions.

Interestingly, the function of BAP1 in stimulating transcription is comparable to that of well-known general co-activators such as SMARCB1 and CREBBP, not only to ensure steady-state gene transcription of hundreds of genes but also in response to stimulus as exemplified by RA treatment. Hence, efficient transcriptional stimulation entails the action of enzymatic activities that converge at creating a permissive chromatin environment, either through histone eviction and/or repositioning (e.g., SMARCB1), reduction of histone charge through acetylation (e.g., CREBBP) or removal of repressive chromatin marks (e.g., BAP1). Interestingly, these different activities can be needed together or separately, as suggested by our observation that SMARCB1, CREBBP, and BAP1 regulate common, as well as specific sets of genes. As BAP1.com generally colocalizes with the transcriptional machinery, we speculate that while present at most transcribed regions, BAP1.com impacts gene expression selectively depending on the chromatin environment. Further investigation will be necessary to decipher what determines the transcriptional response to BAP1 deletion. It will also be interesting to investigate what controls BAP1.com targeting to transcribed regions. We envision that the ASXL proteins, through their PHD finger, a domain that can potentially bind methylated lysine or arginine residues^48^, could participate in reading post-translational marks associated with active transcription. Our work provides insight into the function of BAP1.com and paves the way for novel strategies to target tumors harboring alterations in this chromatin-modifying complex.

## Methods

### Cell lines

HAP1 cells were kindly provided by T. Brummelkamp and cultured in Iscove’s Modified Dulbecco’s Medium media supplemented with 10% fetal bovine serum (FBS) and 1% l-glutamine. HeLa-S3 cells were kindly provided by S. Ait-Si-Ali. They were cultured in adherence in Dulbecco’s modified Eagle’s media (DMEM) supplemented with 10% FBS and 1% l-glutamine. Non-adherent culture of HeLa cells was performed in DMEM supplemented with 5% FBS and 1% l-glutamine following guidelines from Nakatani and Ogryzko^49^. SF9 cells were cultured in SF-900 II serum-free medium (Invitrogen) supplemented with 5% FBS, 1% penicillin/streptomycin (Invitrogen) and Amphotericin B at 28 °C. All cell lines were tested for mycoplasma contamination on a regular basis.

### Constitutive KOs in HAP1 cell line

Mutations of *BAP1*, *ASXL1*, *ASXL2, EZH2, RING1A*, and *SMARCB1* in HAP1 cells were performed using CRISPR/CAS9 technology using the strategy described in^34^. Briefly, a STOP cassette containing a antibiotic resistance gene followed by a polyadenylation sequence from SV40 was inserted by homologous recombination in intronic or exonic sequences of the target genes. Intronic targeting vectors include the EN2 splice acceptor sequence for proper splicing of the antibiotic resistance gene. Antibiotic resistance clones were then picked in 96-well plates and genotyped. KOs were validated by RT-qPCR and western blot when the corresponding antibody was available. The selected clones were thus used as constitutive KOs, using the parental cell line as a control in all experiments. *RING1B* and *CREBBP* KO HAP1 cells were purchased from Horizon Discovery.

### Stable expression in HeLa cells

For mass spectrometry analysis of Flag-tagged BAP1, ASXL1, ASXL2, and KDM1B in HeLa cells, complementary DNAs (cDNAs) encoding the different proteins were first subcloned in pRev retroviral plasmid (gift from S. Ait-Si-Ali), downstream a 2xFlag-2xHA sequence and upstream an internal ribosome entry site sequence followed by CD25 cDNA. Retroviruses were produced by transfection of a 293 Phoenix cell line (gift from S. Ait-Si-Ali) and HeLa-S3 cells were infected by incubation with viral supernatants for 3 h at 37 °C. Infected cells were then selected by fluorescence activated cell sorting against CD25 expression using CD25-FITC antibody and following manufacturer’s instructions (BD Biosciences 553866). Expression was assessed by western blot analysis of nuclear extracts.

### Rescue experiment (BAP1 WT and C91S)

Reintroduction of wild-type or enzymatically dead (C91S) BAP1 was performed by infection of BAP1 KO cells with a pBABE retrovirus^50^. Production of retroviral particles was performed in 293T cells. Transduction was performed by incubating the cells with viral particles mixed with Polybrene (final concentration, 8 μg/ml) for 3 h at 37 °C and subsequently selected with puromycin.

### Proliferation assays

In all, 10,000 cells were plated in six-well plates in triplicates and counted every 24 h over 4 days using a Vi-cells counter (Beckman-Coulter).

### RT-qPCR

Total RNA was isolated using Trizol-Chloroform extraction and isopropanol precipitation. cDNA was synthesized using High Capacity cDNA RT kit (4368814-Applied Biosystems) and quantitative PCR was performed with technical triplicate using SYBR green reagent (Roche) on a ViiA7 equipment (Applied Biosystems). At least two independent experiments (biological replicates) were performed for each assay and RT negative controls were always included. Primer sequences for qPCR analysis are provided in Supplementary Table 1.

### RNA sequencing

RNA sequencing were performed for two independent biological replicates for each condition. In total, 100-bp single-end reads were generated for the RA analysis and 100-bp paired-end reads for all other samples using the HiSeq 2500 platform. Raw reads were trimmed with cutadapt (1.12;^51^) using the Trim Galore! (0.4.4; bioinformatics.babraham.ac.uk) wrapper (default settings) and subsequently aligned to the complete human ribosomal RNA sequence with bowtie (1.2;^52^). Reads that did not align to rRNA were then mapped to the human reference genome (GRCh37/hg19) and gene counts generated with STAR (2.5.2b;^53^) using the following parameters: --quantMode GeneCounts --outSAMtype BAM SortedByCoordinate --runMode alignReads --outFilterMismatchNmax 6 --outFilterMultimapNmax 20 --outSAMmultNmax 20 --outSAMprimaryFlag OneBestScore. Counts were generated using properly paired (for paired-end data) and uniquely mapped reads that overlap the exon boundaries of each gene. More than 94% of reads mapped uniquely for all paired-end sequencing samples and >92% for single end. Per sample read counts are provided in Supplementary Table 2.

BAM files for the CREBBP-KO and HAP1-WT samples were obtained from the Institut de Cancérologie Gustave Roussy and gene counts for these samples were generated using featureCounts (1.5.1;^54^) with the following parameters: -C -p -s 2 -T 8 -F GTF -t exon. Raw reads were trimmed as part of bcl2fastq for Illumina adapters and aligned with RSEM (1.2.25;^55^) and Bowtie2 (2.2.6;^52^) to GRCh37/hg19 using default parameters. Total uniquely mapping reads were calculated using RSeQC (2.6.4;^56^).

The reference FASTA was downloaded from UCSC (http://hgdownload.cse.ucsc.edu/goldenPath/hg19/) and the annotation (GTF) file from gencodegenes.org (comprehensive gene annotation Release 19/GRCh37.p13).

### Differential expression analysis

Genes were filtered to include those with counts per million (CPM) > 0.5 in at least two samples. Raw count data were transformed to log2-CPM and normalized with the TMM method using edgeR (3.18.1; Robinson 2010 and McCarthy 2012). A linear model was fit to the normalized expression values for each gene and empirical Bayes statistics were computed for each KO versus wild-type or rescue with limma (3.32.7; Ritchie, 2015). DE genes were identified from the linear fit after adjusting for multiple comparisons and filtered to include those with FDR < 0.05 and absolute logFC > 1.

GO enrichment analysis was performed with goseq (1.28.0) using the Wallenius method to calculate a probability weighting function for top differential genes as a function of a gene’s median transcript length. GO terms with FDR < 0.01 were collapsed using REVIGO^57^.

### CREBBP, SMARCB1, BAP1 analysis

Raw data for SMARCB1 and WT were downloaded from GEO (GSE75515). RNA-seq analysis was performed as described previously. Only genes common to all datasets were used in the analysis. Significance of overlap for significantly upregulated and downregulated genes between all three KOs was determined applying a one-sided Fisher’s exact test (alternative hypothesis = greater).

### Chromatin immunoprecipitation

For ChIPs experiment, cell confluence and amount of starting material were kept constant by plating defined number of cells 2 days before cross-linking. Briefly, cells were fixed in 1% formaldehyde for 10 min at room temperature, quenched by adding glycine to a final concentration of 0.125 M, rinsed with phosphate-buffered saline (PBS) and resuspended in buffer LB1 (Hepes-KOH pH 7.5 50 mM, NaCl 140 mM, EDTA 1 mM, glycerol 10%, NP-40 0.5%, Triton X-100 0.25% + Protease inhibitors). Cells were rocked at 4 °C for 10 min, pelleted and resuspended in buffer LB2 (NaCl 200 mM, EDTA 1 mM, EGTA 0.5 mM, Tris pH 8 10 mM + Protease inhibitors), pelleted again and resuspended in buffer LB3 (EDTA 1 mM, EGTA 0.5 mM, Tris pH 8 10 mM + Protease inhibitors). Sonication was performed on a Bioruptor (Diagenode), 0.5% *N*-lauroyl-sarcosine added and after rocking at room temperature for 10 min supernatant was kept. For the immunoprecipitation, chromatin (10 μg) was incubated antibodies (around 2 μg) overnight in presence of 1% Triton and 0.1% sodium deoxycholate. Beads blocked with bovine serum albumin were added the day after and incubated at 4 °C for 3 h before processing to the washes in RIPA buffer six times (50 mM Hepes pH 7.6, 10 mM EDTA, 0.7% DOC, 1% NP-40, 0.5 M LiCl + Protease inhibitors) and once in buffer TEN (10 mM Tris pH 8.0, 1 mM EDTA, 50 mM NaCl). Elution was done in buffer TES (50 mM Tris pH 8.0, 10 mM EDTA, 1% sodium dodecyl sulfate), before reversing the crosslink overnight and incubating the samples successively with RNAse A and proteinase K prior to phenol/chloroform/isoamyl-alcohol DNA extraction. ChIPs were analyzed by qPCR using the primers described in Supplementary Table 1.

### ChIP sequencing

In total, 100-bp single-end reads were generated using the HiSeq2500 platform. Reads were mapped to the human reference genome (GRCh37/hg19) with Bowtie2 (2.2.9) using default parameters. PCR duplicates were removed with Picard Tools MarkDuplicates (1.97; http://broadinstitute.github.io/picard). Total uniquely mapping reads were calculated using RSeQC bam_stat.py (2.6.4). BAM files were filtered to exclude common artifact regions (merged consensus artifact regions: http://mitra.stanford.edu/kundaje/akundaje/release/blacklists/hg19-human/). Reads were counted in bins of length 25, RPKM normalized, and converted to bigWig format using DeepTools bamCoverage (2.4.1) for all heatmaps.

Scores upstream and downstream of transcription start sites (TSSs) were computed from normalized bigWig files with deepTools computeMatrix (2.4.1) using reference-point mode. TSS plots were generated with deepTools plotHeatmap (2.4.1).

To identify regions that gain or lose H2AK119ub1, differential analysis was performed using SICER (1.1) between the BAP1 KO and WT H2AK119ub1 using random background to determine statistically enriched regions. To assess the window and gap sizes used in the analysis, we plotted the aggregate score versus the gap size for window sizes of 200, 400, 600, and 800 and gap sizes 1w to 5w. Increasing the window size to 800 showed saturation near a gap of 4w. A gap size of 2400 (3w) corresponded with a score that was sufficiently close to saturation and was chosen for the final analysis. For gap sizes 1w up to 5w, BAP1 KO showed an average of 3.6× more H2AK119ub1 enriched regions than the WT. With w = 800 and g = 2400, 12,388 regions (average length = 24,714.2) were significantly increased (E = 1000, L = 0.74) in the H2AK119ub1 BAP1 KO v. WT and 3456 regions (average length = 30,712.9) were significantly decreased.

### PolII, BAP1 and ASXL1 ChIP-seq analysis

Raw data were downloaded from GEO (GSE40723, GSE36027, GSE51673, and GSE31477). FASTQ files were merged for samples with multiple runs and mapping was performed as previously described to hg19 or mm10. bigWig files were generated with deepTools bamCoverage (2.4.1). Artifact regions were excluded and read counts were normalized to log2(sample/input).

Average values 2 kb around the TSS were computed using deepTools multiBigwigSummary and correlation plots for these regions were generated with deepTools plotCorrelation (--corMethod pearson –removeOutliers –skipZeros).

Merged consensus blacklists for hg19 and mm10 were obtained from the Kundaje lab at Stanford University (Stanford, CA, USA).

### Antibodies

BAP1 (1/500 dilution; C-4; sc-28383), FOXK1 (1/500 dilution; G-4; sc-373810), YY1 (1/500 dilution; sc-7341) and RAR-alpha (1/500 dilution; sc-551 × ) antibodies were purchased from Santa-Cruz; FLAG (1/1000 dilution; M2; F1804) was purchased from Sigma; HCFC1 (1/1000 dilution; A301-400A) and RNF2 (1/1000 dilution; A302-869A) antibodies were purchased from Bethyl Laboratories; Lamin B1 (1/3000 dilution; ab16048); RING1A (1/1000 dilution; 2820S), RING1B (1/1000 dilution; D22F2; 5694S) H2AK119ub1 (1/3000 dilution; D27C4; 8240S), H3K27me3 (1/3000 dilution; C36B11, 9733S), H3K4me3 (1/3000 dilution; C42D8, 9751) and H4 (1/3000 dilution; 2935S) antibodies, were purchased from Cell Signaling Technology; H2A.Z (1/1000 dilution; 39113), H2B (1/1000 dilution; 5HH2-2A8; 61037); H2BK120ub (1/1000 dilution; C56; 39623), H3 (1/3000 dilution; C-terminal; 39163), and KDM1B (1/1000 dilution; 61457) antibodies were purchased from Active Motif; H3K4me2 (1/3000 dilution; MCA-MAB10003-100-Ex) antibody was purchased from Cosmo Bio; alpha-Tubulin (1/3000 dilution; 1F4E3; A01410) was purchased from Genscript.

### Histone extraction

Cells were lysed in a hypotonic lysis buffer (10 mM Tris pH 6.8, 50 mM Na_2_SO_4_, 1%Triton X-100, 10 mM MgCl_2_, 8.6% sucrose, and protease inhibitors) and carefully homogenized using a Dounce A homogenizer. After a centrifugation step at 6000 *g* for 10 min at 4 °C, the pellet was washed with 10 mM Tris pH 7.5, 13 mM EDTA and resuspended in ice-cold water. Protein precipitation was performed by addition of sulfuric acid 0.4 N final concentration and 1-h incubation on ice. The samples were centrifugated at 20,000 *g* for 10 min at 4 °C and the histone-containing supernatant collected and neutralized by addition of 0.5 volume of 1.5 M Tris pH 8.8. Quantification was performed by Bradford assay and confirmed by sodium dodecyl sulfate–polyacrylamide gel electrophoresis (SDS-PAGE) stained with Coomassie.

### Nuclear extracts (high salt)

For nuclear extract preparation, cells were incubated with buffer A (10 mM Hepes pH 7.9, 2.5 mM MgCl_2_, 0.25 M sucrose, 0.1% NP-40 and protease inhibitors) for 10 min on ice, centrifuged at 8000 rpm for 10 min, resuspended in buffer B (25 mM Hepes pH 7.9, 1.5 mM MgCl_2_, 700 mM NaCl, 0.1 mM EDTA, 20% glycerol and protease inhibitors), sonicated and centrifuged at 14,000 rpm for 15 min. Uncropped western blot data are provided in Supplementary Figs. 7–14.

### Chromatography analysis

For analysis of endogenous protein profiles in HAP1 cells, nuclear extracts (high salt) were first dialyzed against BP100 (50 mM potassium phosphate pH 6.8, 100 mM NaCl, 1 mM EDTA, 1 mM DTT, protease inhibitors) and clarified by high-speed centrifugation. Samples were then purified by ion exchange chromatography using a HiTrap SP HP 5 ml column (GE Healthcare). Elution was performed by step elution with increasing NaCl concentration. In all, 500 mM elution was concentrated 5× time on centricon (Millipore, cut-off 10 kDa) and analyzed on Superose 6 PC3.2 increase column (GE Healthcare). The native molecular size markers used for column calibration were thyroglobulin (669 kDa), ferritin (440 kDa), and aldolase (158 kDa).

### Mass spectrometry analysis

For mass spectrometry analysis of Flag-tagged constructs overexpressed in HeLa cells, 100 mg of nuclear extracts were used. Nuclear extracts were first dialyzed in BC250 (50 mM Tris pH 8.0, 250 mM KCl, 1 mM EDTA, 10% Glycerol and protease inhibitors). Precipitates were removed by centrifugation and the supernatant was incubated with anti-FLAG M2 affinity gel overnight. The beads were then washed three times with BC250 + 0.05% NP-40 and eluted with 0.2 mg/ml Flag peptide, precipitated with ice-cold acetone and resuspended in 1× Laemmli Sample Buffer. Of note, for mass spectrometry analysis of Flag-tagged BAP1 overexpressed in MP41 cells, 50 mg of nuclear extracts was used and first purified on an ion exchange chromatography HiTrap Q 1 ml column (GE Healthcare).

After IP and elution of enriched proteins, SDS-PAGE (Invitrogen) was used without separation as a cleanup step to remove lipids, metabolites, salts, and denaturing agents from the samples. After colloidal blue staining (LabSafe GEL BlueTM GBiosciences), four gel slices were excised and proteins were reduced with 10 mM DTT prior to alkylation with 55 mM iodoacetamide. After washing and shrinking the gel pieces with 100% MeCN, in-gel digestion was performed using trypsin/Lys-C (Promega) overnight in 25 mM NH_4_HCO_3_ at 30 °C.

Peptides were extracted and analyzed by nano liquid chromatography coupled to tandem mass spectrometry (LC-MS/MS) using an RSLCnano system (Ultimate 3000, Thermo Scientific) coupled to an Orbitrap Fusion mass spectrometer (Q-OT-qIT, Thermo Fisher Scientific). Samples were loaded on a C18 precolumn (300 µm inner diameter × 5 mm; Dionex) at 20 µl/min in 2% MeCN, 0.05% TFA. After a desalting for 3 min, the precolumn was switched on the C18 column (75 μm i.d. × 50 cm, packed with C18 PepMap™, 3 μm, 100 Å; LC Packings) equilibrated in solvent A (2% MeCN, 0.1% HCOOH). Bound peptides were eluted using a two-step linear gradient of 147 min (from 1 to 20% (v/v)) of solvent B (100% MeCN, 0.085% HCOOH) and 65 min (from 20 to 40% (v/v)) of solvent B, at a 400 nl/min flow rate and an oven temperature of 40 °C. We acquired Survey MS scans in the Orbitrap on the 400–1200 m/z range with the resolution set to a value of 120,000 and a 4 × 10^5^ ion count target. Each scan was recalibrated in real time by co-injecting an internal standard from ambient air into the C-trap. Tandem MS was performed by isolation at 1.6 Th with the quadrupole, higher collisional dissociation fragmentation with normalized collision energy of 35, and rapid scan MS analysis in the ion trap. The MS2 ion count target was set to 10^4^ and the max injection time was 100 ms. Only those precursors with charge state 2–7 were sampled for MS2. The dynamic exclusion duration was set to 60 s with a 10 ppm tolerance around the selected precursor and its isotopes. The instrument was run in top speed mode with 3-s cycles.

Data were searched against the uniprot-Human database, using Sequest HT from Proteome Discoverer 1.4 (thermo Scientific). Enzyme specificity was set to trypsin and a maximum of two miss cleavages was allowed. Oxidized methionine, N-terminal acetylation, and carbamidomethyl cysteine were set as variable modifications. The mass tolerances in MS and MS/MS were set to 10 ppm and 0.6 Da, respectively. The resulting files were further processed using myProMS^58^. The Sequest HT target and decoy search results were validated at 1% FDR with Percolator. The mass spectrometry proteomics data have been deposited to the ProteomeXchange Consortium via the PRIDE partner repository with the dataset identifier PXD011808^59^.

### Methylation analysis

Genomic DNA was extracted following manufacturer protocol (DNA easy, Qiagen), treated with RNAse A and RNAse T1, purified again and quantified using nanodrop. One microgram of DNA was then digested by the degradase plus (Zymoresearch), ethanol precipitated and the supernatant was then evaporated on speedvac. Samples were then reconstituted in 10 μl of solution A’ (2% methanol, 0.1% HCOOH), vortex-mixed, centrifuged and transferred to a high-performance liquid chromatography vial for micoLC-MS/MS analysis. Fractions were used directly in solution A’ and analyzed (5 μl) using the RSLCnano system connected to the Orbitrap Fusion mass spectrometer. Sample separation was achieved on a C18 column (4.6 × 100 mm, packed with ZORBAX Eclipse XDB C18, 1.8 µm particles, Agilent Technologies) after 5 min loading in solvent A’, with a linear gradient of 10 min (from 0 to 30% (v/v) of solvent B’ (80% MeCN, 0.1% HCOOH)) at 500 μl/min. Data acquisition was performed in the Orbitrap on the 200–300 m/z range with the resolution set to a value of 240,000 at m/z 200. To determine the intensity of each nucleosides, we extracted from the MS survey of microLC-MS/MS raw files the extracted ion chromatogram (XIC) signal by using the retention time and m/z values of the well-characterized synthetic nucleoside ions using the Xcalibur softwares (manually). XIC areas were integrated in Xcalibur under the QualBrowser interface using the ICIS algorithm.

### Reporting summary

Further information on experimental design is available in the Nature Research Reporting Summary linked to this article.

## Supplementary information

Supplementary Information

Reporting Summary

## Data Availability

ChIP-seq and RNA-seq data that support the findings of this study have been deposited in the Gene Expression Omnibus under the accession codes GSE110133 (ChIP-seq) and GSE110142 (RNA-seq) [https://www.ncbi.nlm.nih.gov/geo/query/acc.cgi?acc = GSE110143]. The raw protomics data are available via ProteomeXchange with identifier PXD011808. Raw data are provided for all western blots in Supplementary Figs. 7–14. All other relevant data supporting the key findings of this study are available within the article and its Supplementary Information files or from the corresponding authors upon reasonable request. A Reporting Summary for this Article is available as a Supplementary Information file.
