# Supplementary material for: BAP1 complex promotes transcription by opposing PRC1-mediated H2A ubiquitylation
Source: Nat Commun. 2019 Jan 21;10:348. doi: 10.1038/s41467-018-08255-x (PMC6341105; doi:10.1038/s41467-018-08255-x)
Supplement: Supplementary file 1 — Supplementary Information [file 41467_2018_8255_MOESM1_ESM.pdf]

SUPPLEMENTARY INFORMATION

BAP1 complex promotes transcription by opposing PRC1-mediated H2A ubiquitylation

Campagne *et al.*

Content:

Supplementary Figure 1
Supplementary Figure 2
Supplementary Figure 3
Supplementary Figure 4
Supplementary Figure 5
Supplementary Figure 6
Supplementary Figure 7 (raw data for Figure 1C)
Supplementary Figure 8 (raw data for Figure 2B and 2D)
Supplementary Figure 9 (raw data for Figure 3A)
Supplementary Figure 10 (raw data for Figure 4B)
Supplementary Figure 11 (raw data for Figure 6A and 6B)
Supplementary Figure 12 (raw data for Figure S1A and S1B)
Supplementary Figure 13 (raw data for Figure S2A, S2B and S2E)
Supplementary Figure 14 (raw data for Figure S5A)

Supplementary Table 1
Supplementary Table 2

Supplementary Methods
Supplementary bibliography

Figure S1

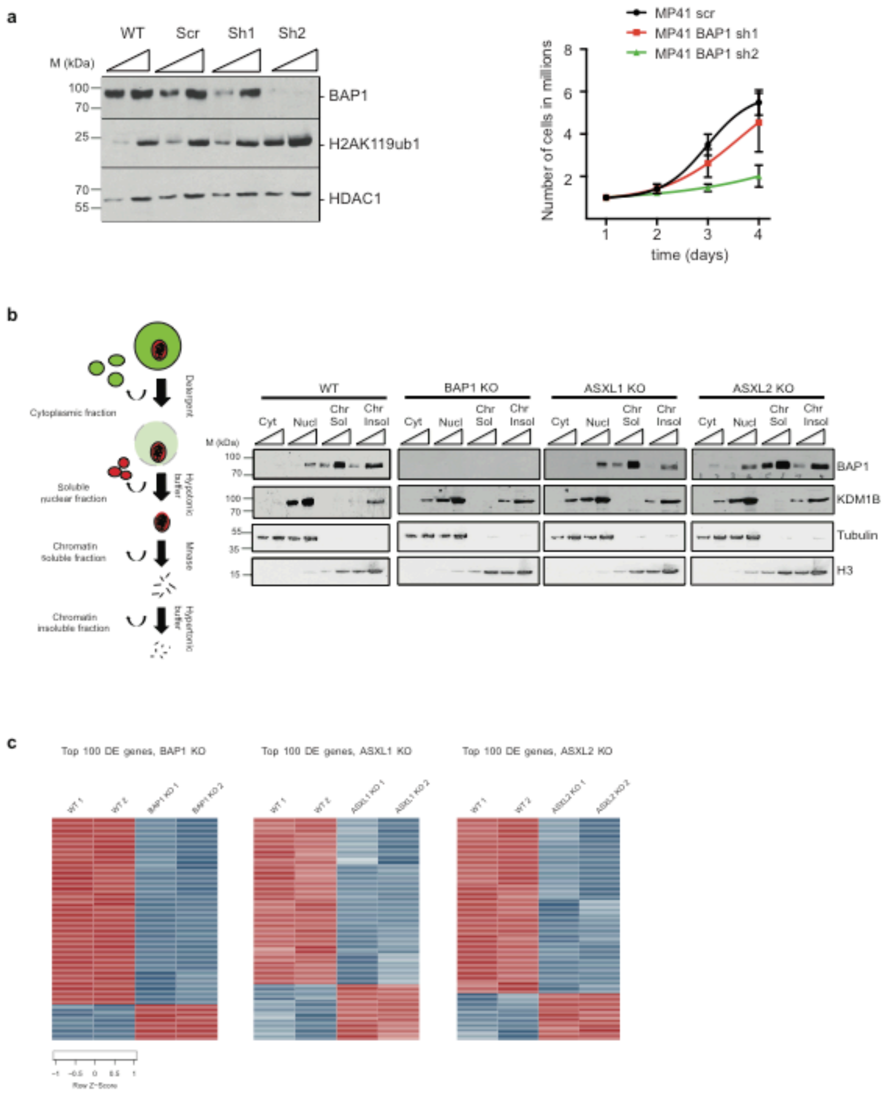

Figure S1: BAP1.com activity

a) RNA interference against *BAP1* in MP41 cells. Left panel: MP41 were infected with lentivirus containing shRNAs and nuclear extracts were analyzed by western blot. Scr = sh-scramble. A two-point titration (1:3 ratio) is shown for each condition. Right panel: proliferation assay in wild type or BAP1-silenced MP41 cells. Figures represent the mean of 3 independent biological replicates. Error bars represent the standard deviation between the replicates. **b)** Left panel: scheme summarizing the fractionation strategy for the isolation of chromatin-interacting proteins. Right panel: Western blots assessing the distribution of BAP1 and KDM1B in the different cellular compartments and chromatin fractions in HAP1 wild type and knockout cells. Tubulin and Histone H3 serve as controls for the cytoplasmic and chromatin associated fractions respectively. Two quantities were loaded for each condition, respecting a 1:3 ratio. **c)** Heatmap of 100 top differentially expressed (DE) genes in *BAP1*, *ASXL1*, and *ASXL2* KO cells. Individual replicates are shown side by side.

Figure S2

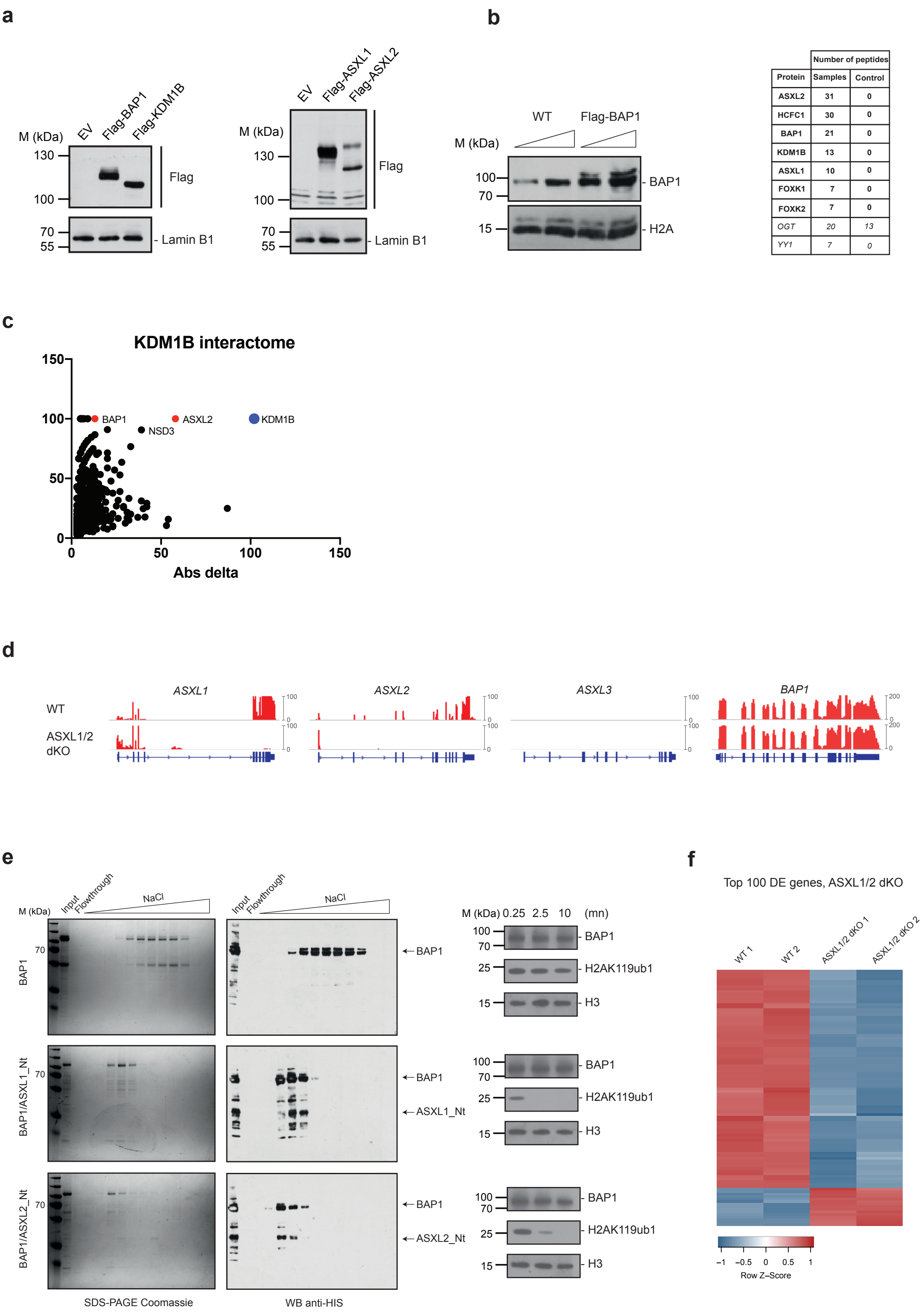

	Number of peptides	
Protein	Samples	Control
ASXL2	31	0
HCFC1	30	0
BAP1	21	0
KDM1B	13	0
ASXL1	10	0
FOXK1	7	0
FOXK2	7	0
OGT	*20*	*13*
YY1	*7*	*0*

Figure S2: BAP1.com composition.

a) Western blot analysis of nuclear extracts from HeLa cells expressing Flag-tagged constructs or empty vector as control. Lamin B1 was used as a loading control. **b)** Left panel: western blot showing overexpression of Flag-BAP1 in MP41 nuclear extracts. H2A was used as a loading control. Right panel: table recapitulating mass spectrometry result of Flag-BAP1 IP as control to control in MP41 cells. **c)** Mass spectrometry analysis of HeLa cells overexpressing FLAG-tagged KDM1B. Graphs represent proteins relative to their absolute and relative delta compared to mass spectrometry analysis of empty vector expressing cells. **d**) Screenshots of RNA-seq on wild-type and ASXL1/2 dKO cells showing expression of ASXL1, ASXL2, ASXL3 (not expressed), and BAP1. **e)** Left panel: coomassie and corresponding western blot analyses showing the anion exchange purification of recombinant BAP1, BAP1+ASXL1_Nt and BAP1+ASXL2_Nt (Mono Q PC 0.1ml). The input is issued from a first Flag IP on whole cell extracts of SF9 expressing the different combinations of recombinant proteins. The asterisk represents a non-specific protein co-IPed with Flag-BAP1. Right panel: representative result of deubiquitinase assay following H2AK119 ubiquitination, western blot for BAP1 serves as a control. **f**) Heatmap of 100 top differentially expressed (DE) genes in *ASXL1/2* dKO cells. Individual replicates are shown side by side.

Figure S3

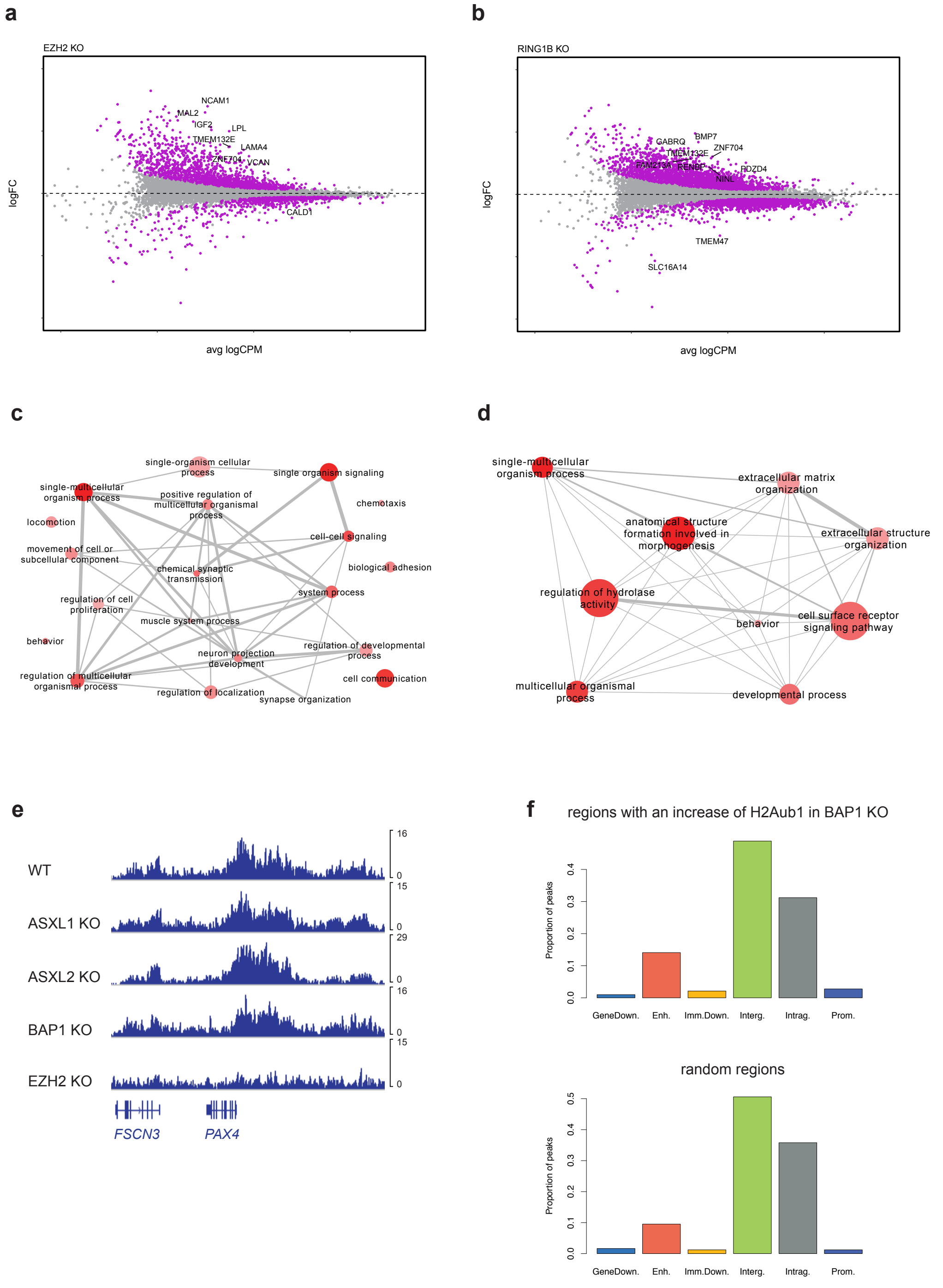

Figure S3: BAP1 versus the Polycomb machinery

a, b) Scatterplots showing log2 fold-change (logFC) expression between wild-type and *EZH2* KO (a) or *RING1B* KO (b) cells as a function of average log2 counts per million (logCPM). Differentially expressed genes (DEGs) are highlighted in magenta. **c, d**) Gene ontology analysis of DEGs in *EZH2* KO (c) and *RING1B* KO (d) cells. **e**) Screenshot showing H3K27me3 enrichment at the *PAX4* locus in the different conditions indicated on the left. **f**) Annotation of regions that gain H2AK119ub1 upon *BAP1* KO (top panel) versus a random permutation of the same regions over the human genome (bottom panel).

Figure S4

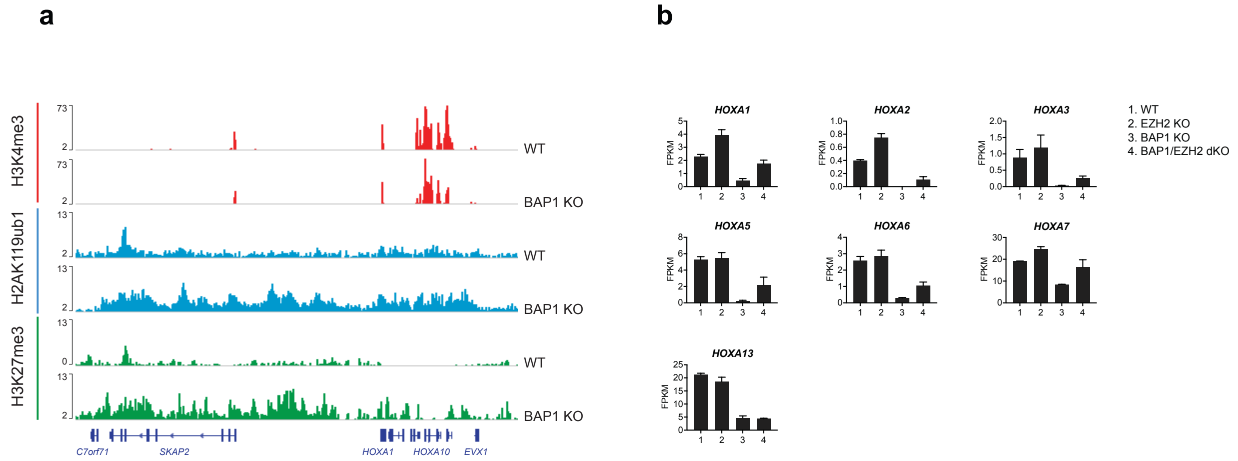

Figure S4: BAP1 versus PRC2

a) Screenshot showing enrichment for H3K4me3, H2AK119ub1 and H3K27me3 at the HOXA cluster in wild-type and *BAP1* KO cells. **b**) Expression of the individual HOXA genes represented as column bar graphs across wild-type, *EZH2*, *BAP1* and *BAP1/EZH2* KO conditions detected from corresponding RNA-seq data. Mean + SD, n=2.

Figure S5

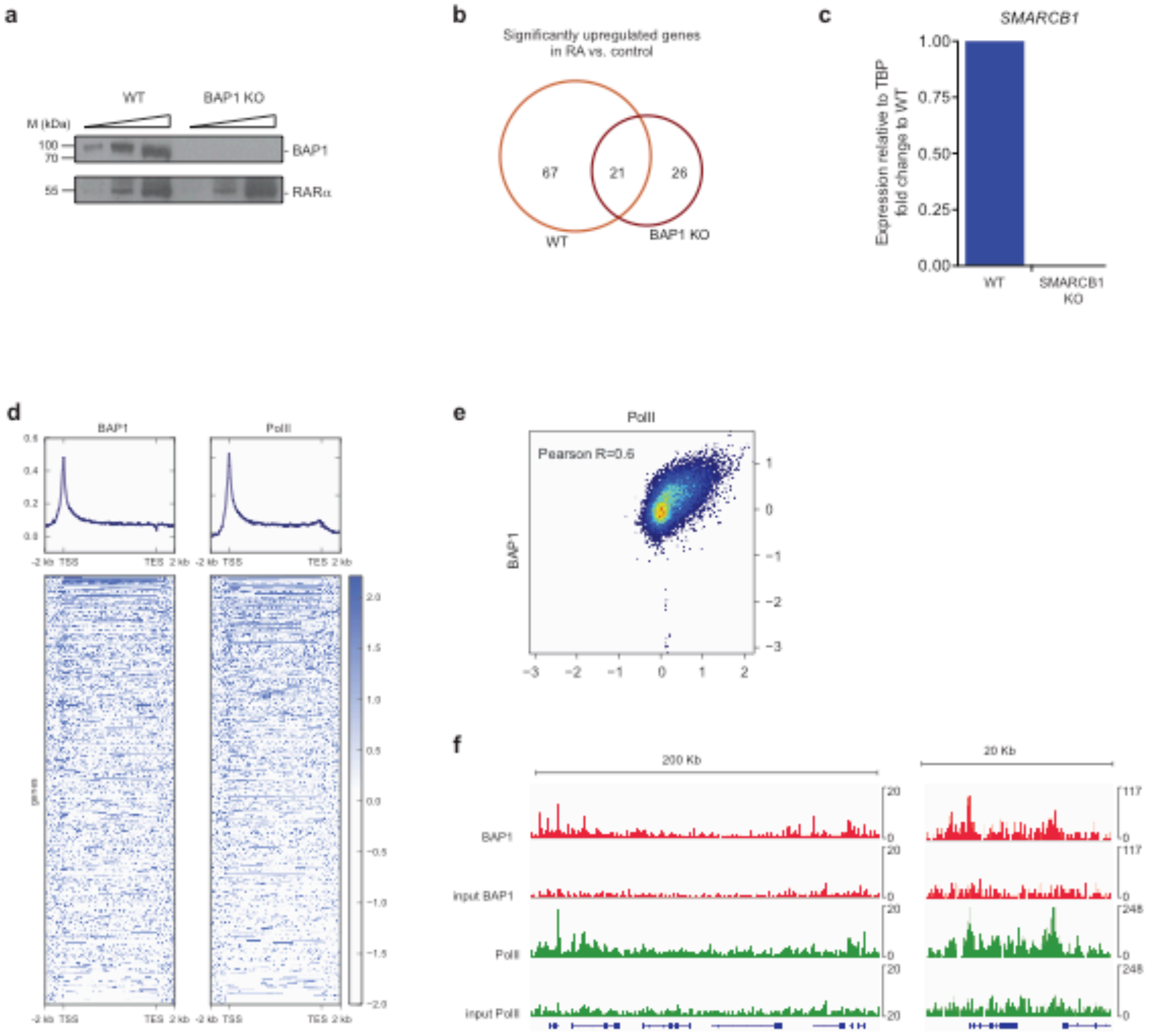

Figure S5: BAP1 and transcription

a) Titration of nuclear extracts from wild-type or *BAP1* KO HAP1 cells analyzed by western blot and probed with antibodies recognizing either BAP1 or RARα. **b**) Venn diagram showing the overlap between RA-regulated genes in wild-type and BAP1 KO cells. **c)** RT-qPCR analysis SMARCB1 expression in the different in wild-type and SMARCB1 KO HAP1 cells. Mean ± SD. n = 3. **d)** Heatmaps showing BAP1 and RNA PolII along gene bodies of all annotated genes from TSS to termination end site (TES) scaled to an equivalent 10 kb window and including 2 kb upstream and downstream of TSS and TES respectively. Corresponding average profiles are plotted on top of each heatmap. **e**) Scatterplot showing PolII versus BAP1 enrichment around the TSS (-/+ 2 kb) at all annotated genes in mouse Bone Marrow-Derived Macrophages. Pearson correlation coefficient is displayed. **f**) Screenshots of BAP1 and RNA PolII enrichment at representative regions. The input displayed below each corresponding ChIP-seq experiment.

Figure S6

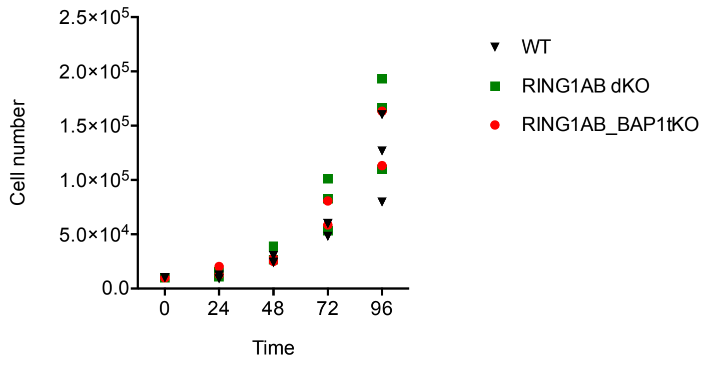

Figure S6: BAP1 versus PRC1.

Proliferation curve of wild-type, *RING1A/B* dKO and *RING1A/B*;*BAP1* KO HAP1 cells. n = 3.

Figure S7

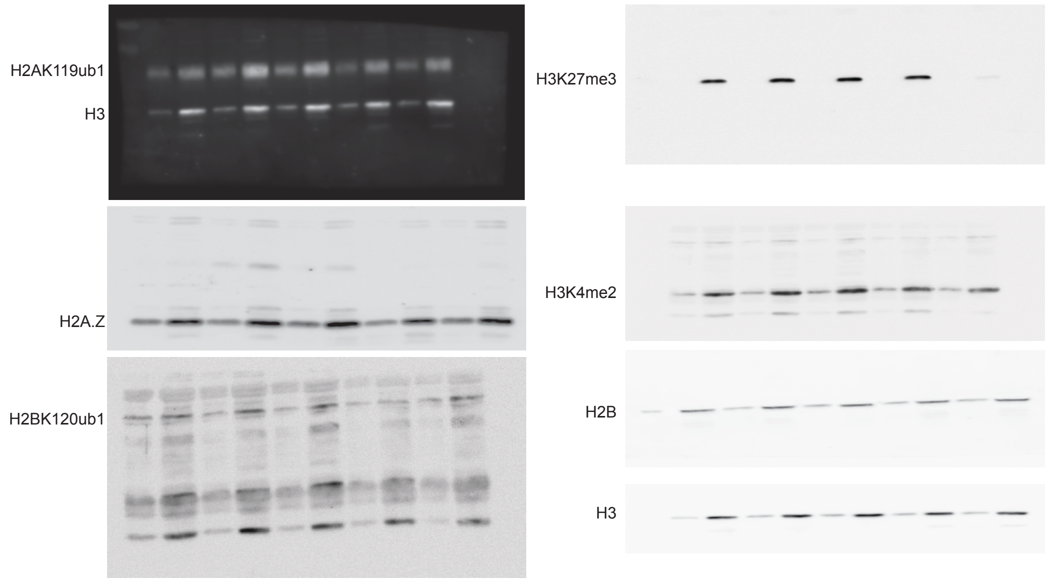

Figure S7: raw data for Figure 1C

Figure S8

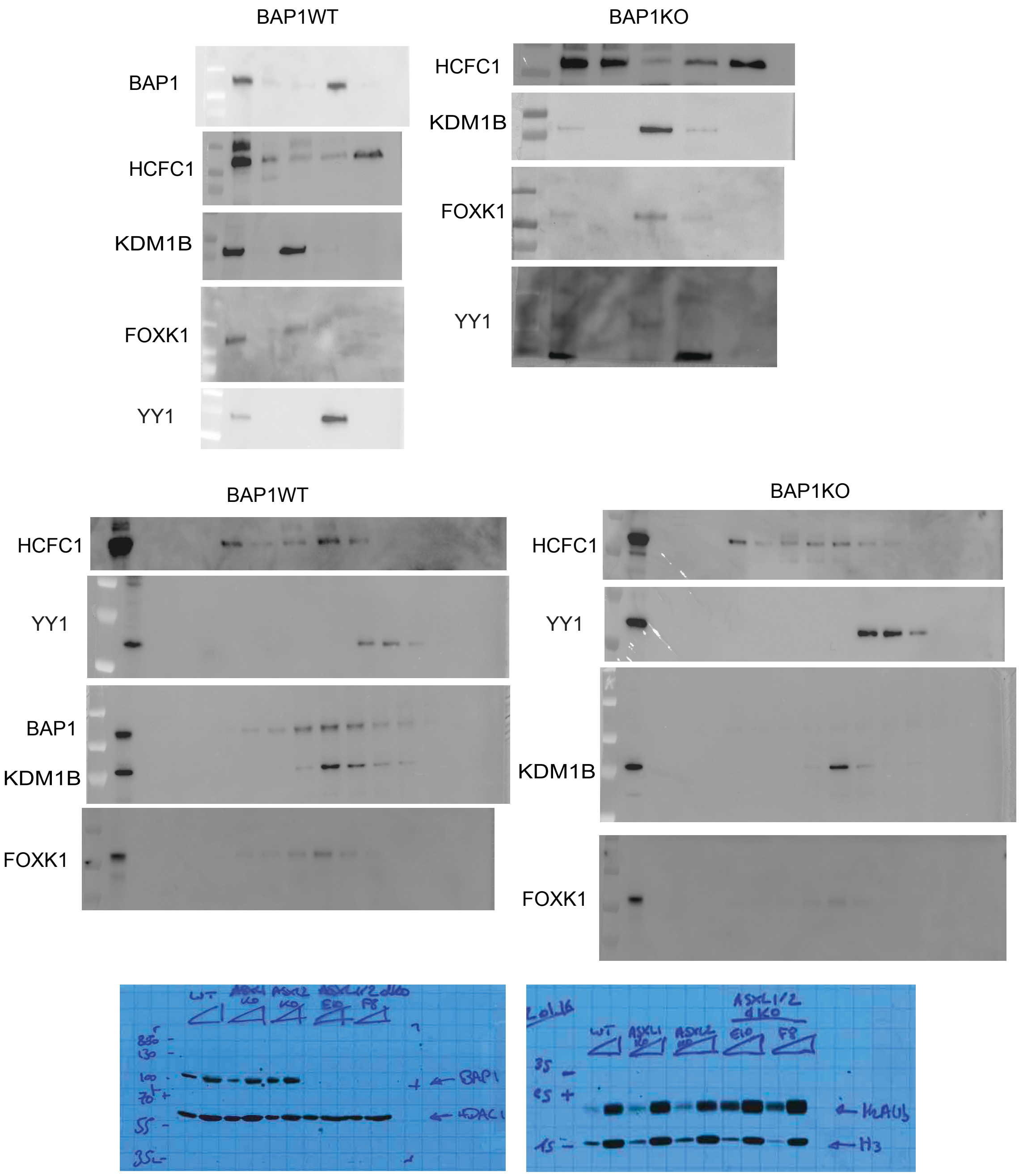

Figure S8: raw data for Figure 2B and 2D

Figure S9

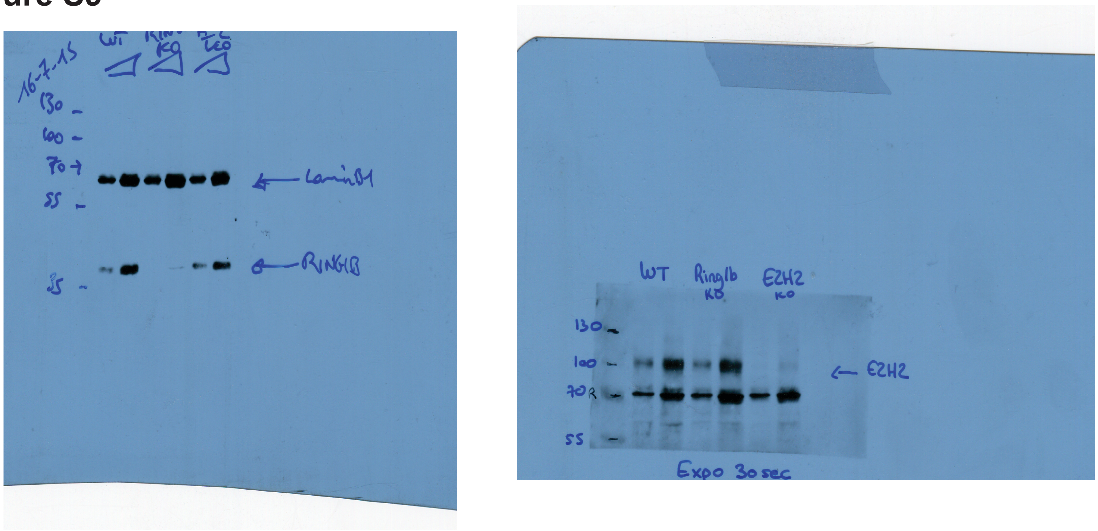

Figure S9: raw data for Figure 3A

Figure S10

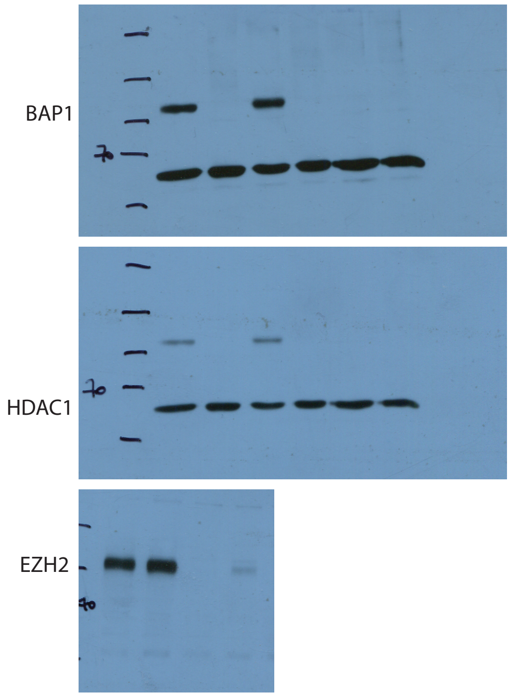

Figure S10: raw data for Figure 4B

Figure S11

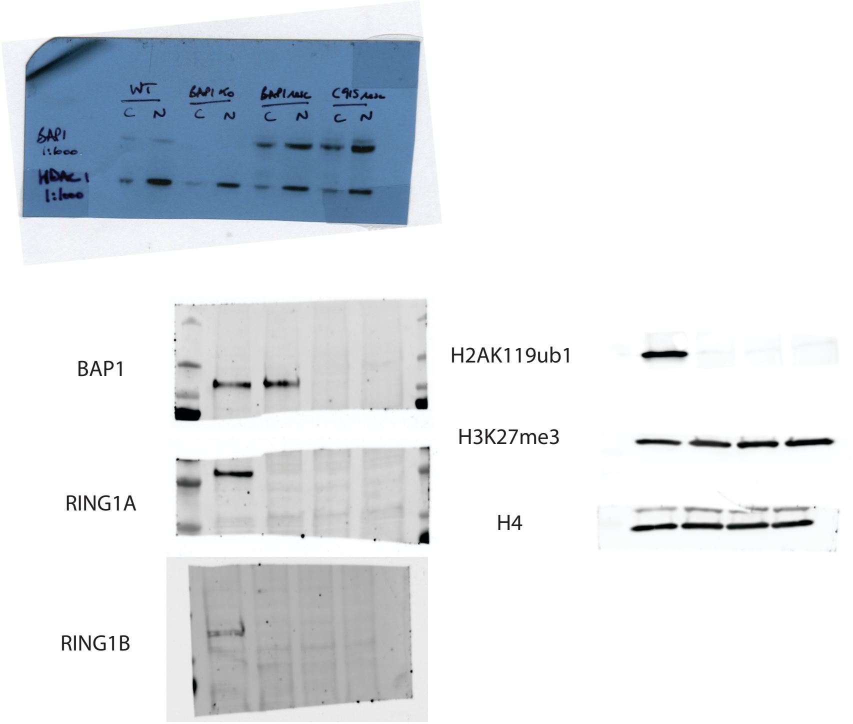

Figure S11: raw data for Figure 6A and 6B

Figure S12

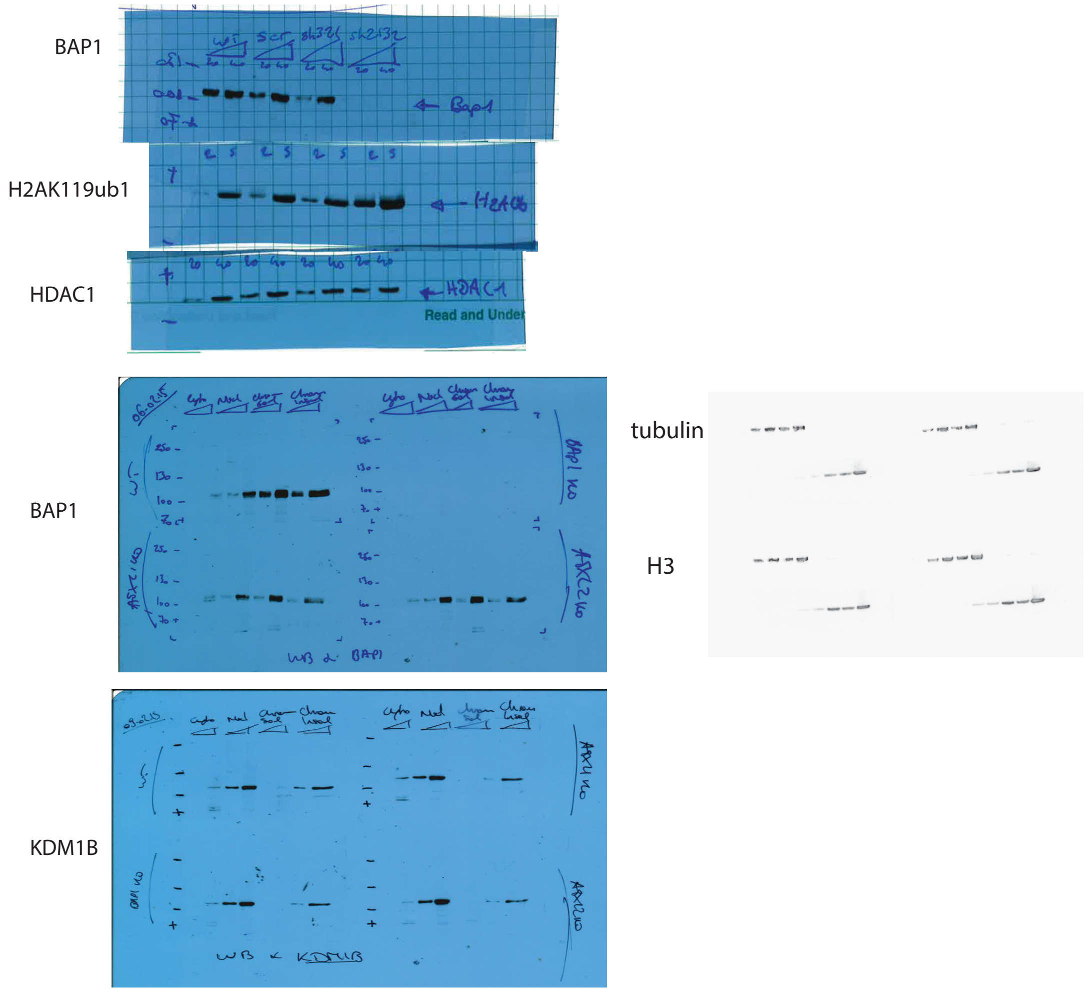

Figure S12: raw data for Figure S1A and S1B

Figure S13

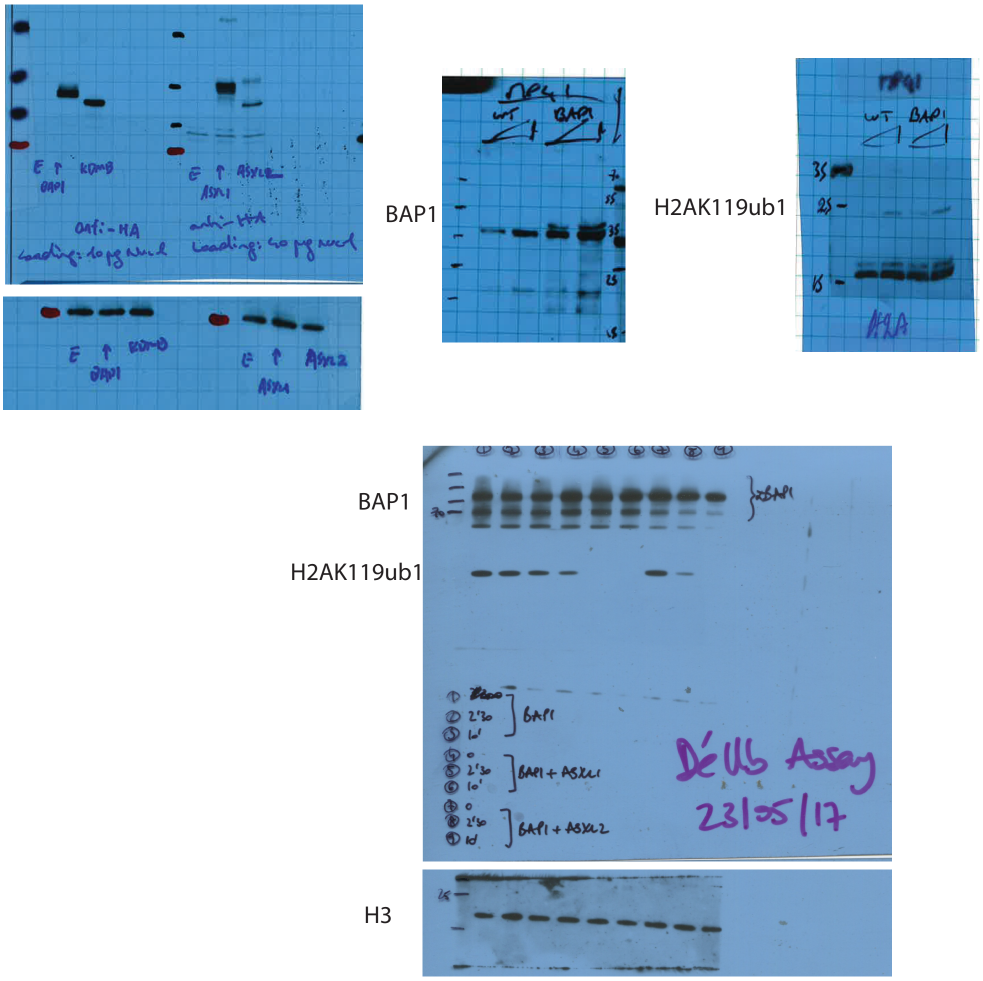

Figure S13: raw data for Figure S2A, S2B and S2E

Figure S14

BAP1

RAR alpha

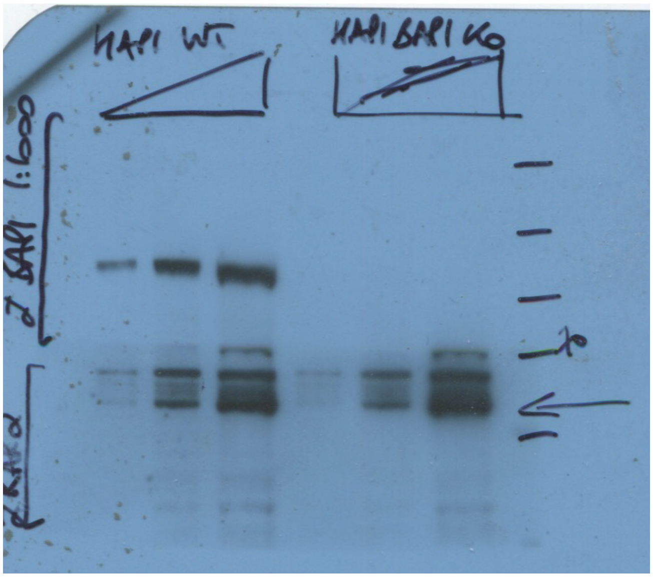

Figure S14: raw data for Figure S5A

primer	sequence
TBP_FW	GTTCTGGGAAAATGGTGTGC
TBP_REV	GCTGGAAAACCCAACTTCTG
SMARCB1_FW	AGACCAGCGCGTCATCATCA
SMARCB1_REV	CAGCTCCGAGCACAGCTTCA
EZH2_FW	TTTTTGCCAAGAGAGCCATC
EZH2_REV	TCGATGCCGACATACTTCAG
ASXL1_FW	AAAGCCACAGCCCACTAAAG
ASXL1_REV	AGTGGGCTGACCTTTAACCAC
ASXL2_FW	GACAGAATCCAGGTGCGAAAAG
ASXL2_REV	TTCTGGCTCCTGTTCTGTTAGG
BAP1_FW	CCACAAGTCTCAAGAGTCACAG
BAP1 _REV	CTGCACCATCTGTGTGGTT
RARB_FW	AGCAAGCCTCACATGTTTCC
RARB_REV	CTCTGCACcTTTAGCACTGATG
CYP26A1_FW	AACATTCGCGCCAAGATCTG
CYP26A1_REV	TGCTTTAGTGCcTGCATGTC
S100A11_FW	TCGCTCAGCTCCAACATGGCAA
S100A11_REV	ACAGCAATCAGGGACTCGATGCAC
TNFRSF1A_FW	TGCCAGGAGAAACAGAACACCGT
TNFRSF1A_REV	AGGCACAACTTCGTGCACTCCA
TMSB4X_FW	TCGCTTCGCTTTTCCTCCGCAA
TMSB4X_REV	GCCTGCTTGCTTCTCCTGTTCAATC
DHRS3_FW	CTACTGCACATCCAAAGCGTC
DHRS3_REV	GGGAAACCTGACTCTCATGCC
FAM46A_FW	CTGGACTGCCTGTTGGACTTC
FAM46A_REV	TCCATCGGTCAGAGTCATTGC

Supplementary table 1: Primer sequences used for RT-qPCR analysis

RNA-seq

ID	NAME	TOTAL	MAPPED	DUPLICATION RATE
**B140T5**	A1_1	70241530	69450520	0.15
**B140T6**	A1_2	64937844	64203469	0.14
**B140T7**	A2_1	58673448	57958778	0.15
**B140T8**	A2_2	53059964	52345028	0.13
**B178T11**	ASXL1_2_KO_1	67091976	65946970	0.14
**B178T12**	ASXL1_2_KO_2	54790946	54118638	0.13
**B140T3**	B_1	63357286	62600276	0.15
**B140T4**	B_2	57635620	56811726	0.13
**A733T146**	B_EC2dKOcontrol_1	24281347	23941732	0.63
**A733T151**	B_EC2dKOcontrol_2	28806113	28311712	0.65
**A733T143**	B_EC2dKORA24h_1	31408623	31045728	0.65
**A733T152**	B_EC2dKORA24h_2	26898827	26484207	0.64
**B178T7**	BAP1_EZH2_KO_1	65846202	65017975	0.16
**B178T8**	BAP1_EZH2_KO_2	58402232	57109340	0.15
**B178T3**	BAP1_KO_1	54022002	53175371	0.13
**B178T4**	BAP1_KO_2	55476234	54567352	0.17
**B178T13**	BAP1_KO_3	59298136	58449034	0.14
**B178T14**	BAP1_KO_4	59040088	57956440	0.14
**B178T15**	BAP1_rescue_2	56640210	55663882	0.15
**B178T16**	BAP1_rescue_3	59806914	58635322	0.16
**A733T142**	BAP1KOcontrol_1	29799276	29478507	0.61
**A733T149**	BAP1KOcontrol_2	24683840	24423284	0.61
**A733T141**	BAP1KORA24h_1	26447921	26155065	0.59
**A733T150**	BAP1KORA24h_2	23339424	23041865	0.61
**B140T9**	E_1	74148166	72896586	0.16
**B140T10**	E_2	60561476	59849531	0.13
**B178T9**	EZH2_BAP1_KO_1	62641432	61827211	0.14
**B178T10**	EZH2_BAP1_KO_2	51817994	51068947	0.13
**B178T5**	EZH2_KO_1	59706336	58712569	0.13
**B178T6**	EZH2_KO_2	62309352	61359293	0.14
**B140T11**	R_1	66889238	65806493	0.13
**B140T12**	R_2	63433256	62060800	0.13
**B140T1**	W_1	66790126	65956987	0.15
**B140T2**	W_2	56378590	55652212	0.14
**B178T1**	WT_1	48609558	47885617	0.13
**B178T2**	WT_2	54067260	53298331	0.15
**A733T144**	WTcontrol_1	24508360	24275817	0.62
**A733T147**	WTcontrol_2	26780729	26551175	0.63
**A733T145**	WTRA24h_1	24723651	24443565	0.61
**A733T148**	WTRA24h_2	27751900	27449448	0.63

ChIP-seq

ID	NAME	TOTAL	MAPPED	DUPLICATION RATE
**B139C13**	A1_K27_1	44631872	43672079	0.05
**B139C14**	A1_K27_2	43962771	42998869	0.05
**B139C15**	A1_K4_1	43736044	42624486	0.08
**B139C16**	A1_K4_2	58785798	57249240	0.07
**B139C17**	A1_Ub_1	61794710	60499768	0.05
**B139C18**	A1_Ub_2	61923203	60577687	0.05
**B139C19**	A2_K27_1	41963427	41224761	0.06
**B139C20**	A2_K27_2	43886633	43283990	0.06
**B139C21**	A2_K4_1	37705765	37064784	0.3
**B139C22**	A2_Ub_1	42531701	41783297	0.04
**B139C23**	A2_Ub_2	47786002	46990575	0.05
**B139C7**	B1_K27_1	37654429	36784099	0.06
**B139C8**	B1_K27_2	43611916	42610006	0.06
**B139C9**	B1_K4_1	46233393	45066997	0.09
**B139C10**	B1_K4_2	43836218	42622688	0.07
**B139C11**	B1_Ub_1	58753665	57660167	0.05
**B139C12**	B1_Ub_2	49537001	48526449	0.04
**B139C24**	E2_K27_1	38119713	37044115	0.07
**B139C25**	E2_K27_2	43338116	42150861	0.09
**B139C26**	E2_K4_1	43621734	42502791	0.09
**B139C27**	E2_K4_2	52617910	51236451	0.07
**B139C28**	E2_Ub_1	47589844	46541422	0.05
**B139C29**	E2_Ub_2	42187354	41171030	0.05
**B139C30**	RB_K27_1	39666722	38921385	0.08
**B139C31**	RB_K27_2	36743282	36222362	0.06
**B139C32**	RB_K4_1	32513561	31984090	0.3
**B139C33**	RB_Ub_1	34491103	33826182	0.04
**B139C34**	RB_Ub_2	34774245	34164733	0.05
**B139C1**	WT_K27_1	37461281	36604007	0.05
**B139C2**	WT_K27_2	36936404	36074797	0.05
**B139C3**	WT_K4_1	38014632	37022611	0.08
**B139C4**	WT_K4_2	38243693	37203001	0.07
**B139C5**	WT_Ub_1	41263065	40368888	0.05
**B139C6**	WT_Ub_2	39021850	38084567	0.05

Supplementary table 2: Mapping statistics for RNA-seq and ChIP-seq samples

Supplementary methods

Cell lines

MP41 cells were kindly provided by Dr S. Roman-Roman and cultured in RPMI media supplemented with 10% FBS and 1% L-Glutamine (Invitrogen).

BAP1 knockdown

shRNA sequences targeting BAP1 were retrieved from the following publication [1] and cloned into pLKO.1 vector from addgene.
The target sequences are:

shBAP1.321	CGUCCGUGAUUGAUGAUGA
shBAP1.2132	GAGUUCAUCUGCACCUUUA

Supplementary bibliography

1. Machida, Y.J., Machida, Y., Vashisht, A.A., Wohlschlegel, J.A. & Dutta, A. The deubiquitinating enzyme BAP1 regulates cell growth via interaction with HCF-1. *J Biol Chem* **284**, 34179-88 (2009).
